# Supplementary material for: Mannose-binding lectin and complement mediate follicular localization and enhanced immunogenicity of diverse protein nanoparticle immunogens
Source: Cell Rep. Author manuscript; Available in PMC 2022 Feb 1. (PMC8805147; doi:10.1016/j.celrep.2021.110217)
Supplement: 1 [file NIHMS1771086-supplement-1.pdf]

Cell Reports, Volume *38*

Supplemental information

Mannose-binding lectin and complement mediate follicular localization and enhanced immunogenicity of diverse protein nanoparticle immunogens

Benjamin J. Read, Lori Won, John C. Kraft, Isaac Sappington, Aereas Aung, Shengwei Wu, Julia Bals, Chengbo Chen, Kelly K. Lee, Daniel Lingwood, Neil P. King, and Darrell J. Irvine

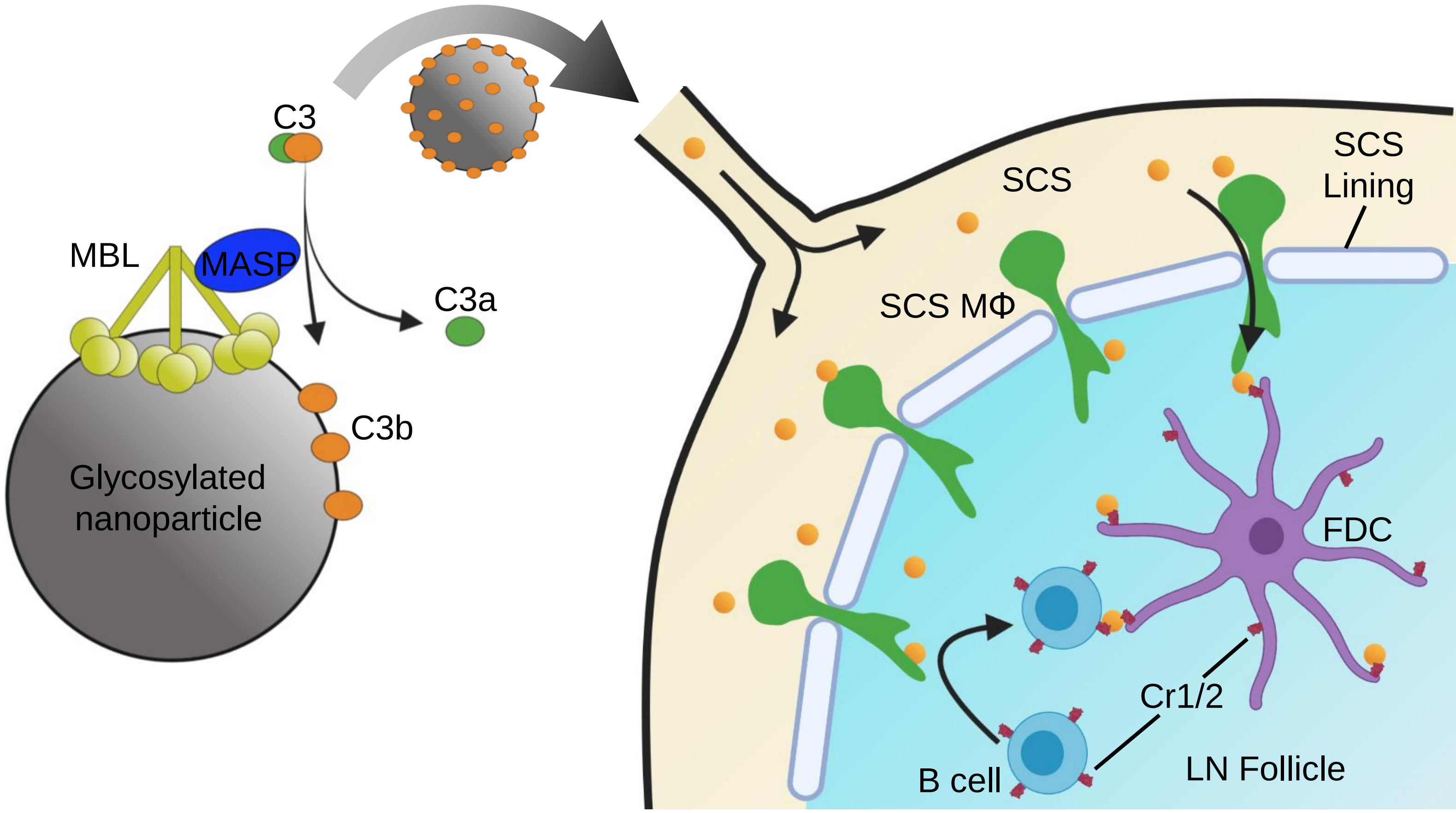

Figure S1. Schematic of MBL-mediated nanoparticle trafficking pathway. Related to Figure 1. Heavily glycosylated nanoparticulate antigens are bound by MBL following injection. Upon MBL binding, MBL-associated serine proteases (MASPs) become active and either directly or indirectly cleave C3 into components C3a and C3b, the latter of which opsonizes the nanoparticle. Opsonized nanoparticles enter the lymph node from afferent lymphatic vessels into the subcapsular sinus (SCS) and are bound by subcapsular sinus macrophages (SCS MΦ), which transport particles to the interior of the node through translocation and/or transcytosis. The nanoparticles are then transported to follicular dendritic cells (FDCs) by B cells, via cognate B cell receptor recognition or noncognate complement receptor (Cr1 and Cr2) binding, or by direct binding of an FDC dendrite, where they are captured and displayed by Cr1 and Cr2.

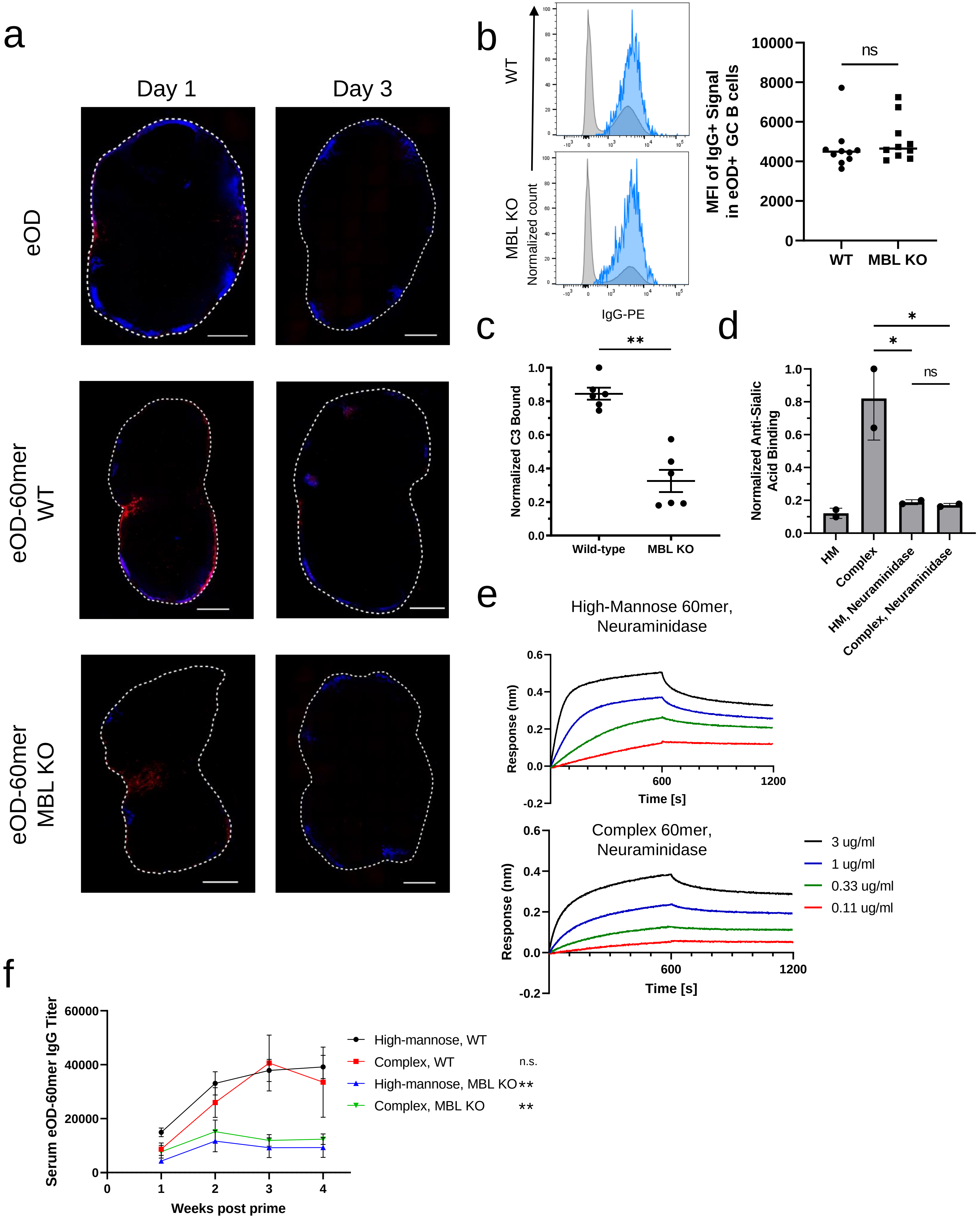

a
Day 1
Day 3
eOD
eOD-60mer WT
eOD-60mer MBL KO
b
WT
MBL KO
Normalized count
IgG-PE
MFI of IgG+ Signal in eOD+ GC B cells
ns
WT
MBL KO
c
Normalized C3 Bound
**
Wild-type
MBL KO
d
Normalized Anti-Sialic Acid Binding
*
*
ns
HM
Complex
HM, Neuraminidase
Complex, Neuraminidase
e
High-Mannose 60mer, Neuraminidase
Response (nm)
Time [s]
Complex 60mer, Neuraminidase
3 ug/ml
1 ug/ml
0.33 ug/ml
0.11 ug/ml
f
Serum eOD-60mer IgG Titer
Weeks post prime
High-mannose, WT
Complex, WT
n.s.
High-mannose, MBL KO **
Complex, MBL KO **

Figure S2. Additional lymph node trafficking and characterization of eOD-60mer. Related to Figures 2 and 3. a C57Bl/6 or MBL KO mice (n=5/group) were immunized with 2 µg eOD equivalent AlexaFluor 647-labeled eOD-GT8 60mer or eOD monomer and saponin adjuvant. One or three days post immunization, lymph nodes were snap-frozen and cryosectioned for confocal imaging. Blue, CD35; red, eOD-GT8; scale bars denote 500 µm. **b** C57Bl/6 or MBL KO (n=10/group) were immunized with 2 µg eOD equivalent eOD-GT8 60mer and saponin adjuvant. Draining inguinal lymph nodes were removed 12 days post immunization and analyzed by flow cytometry. Shown are representative histograms of IgG signal MFI among all cells (gray) and antigen-specific GC B cells (blue) and the antigen-specific GC B cell eOD signal MFI from each sample. **c** High-mannose eOD-60mer was incubated for two hours in either 10% fresh wild-type or MBL KO serum (n=6/group), followed by detection with an anti-C3 antibody to determine the degree of C3 deposition. Error bars indicate SEM; **,$p<0.01$ by Mann-Whitney test. **d** High-mannose and complex eOD-60mer were treated with neuraminidase to remove sialic acid residues, which was confirmed via binding of polyclonal anti-sialic acid. n=2/group, *,$p<0.05$ by one-way ANOVA followed by Tukey post hoc test. **e** BLI analysis of high-mannose and complex eOD-60mer treated with neuraminidase binding to murine MBL2 as a function of eOD particle concentration. **f** Serum eOD-60mer specific IgG titers over time in wild-type and MBL KO mice immunized with either high-mannose or complex eOD-GT8 60mer. Error bars indicate SEM; **,$p<0.01$; n.s.=not significant relative to high-mannose WT by one-way ANOVA followed by Tukey post hoc test.

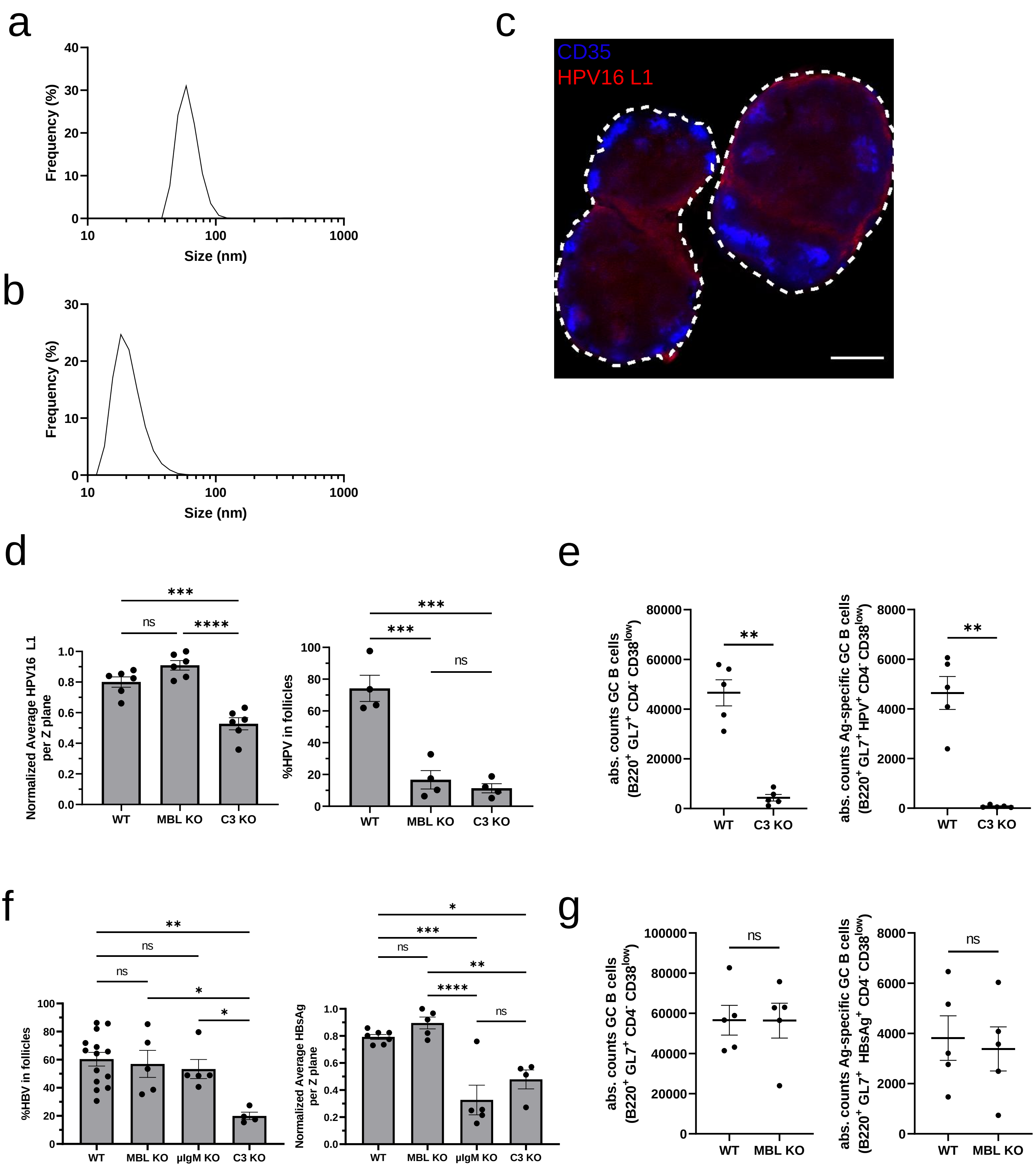
a
Frequency (%)
Size (nm)
b
Frequency (%)
Size (nm)
c
CD35
HPV16 L1
d
Normalized Average HPV16 L1 per Z plane
WT
MBL KO
C3 KO
%HPV in follicles
e
abs. counts GC B cells (B220+ GL7+ CD4- CD38low)
abs. counts Ag-specific GC B cells (B220+ GL7+ HPV+ CD4- CD38low)
f
%HBV in follicles
µIgM KO
Normalized Average HBsAg per Z plane
g
abs. counts GC B cells (B220+ GL7+ CD4- CD38low)
abs. counts Ag-specific GC B cells (B220+ GL7+ HBsAg+ CD4- CD38low)
ns

Figure S3. Additional HPV16 L1 and HBsAg characterization and *in vivo* response data. Related to Figure 5. a-b Hydrodynamic diameters of HPV16 L1 (**a**) and HBsAg (**b**) as determined by dynamic light scattering. **c** C3 KO mice (n=5/group) were immunized with 0.1 µg AlexaFluor 647-labeled HPV16 L1 and saponin adjuvant. 7 days later, lymph nodes were harvested, cleared, and imaged by confocal microscopy. Shown are average intensity Z projections through 360 µm of tissue; shown is staining for CD35 (blue) and antigen (red), scale bars denote 500 µm. **d** Analyses of normalized total HPV16 L1 signal per Z plane of cleared lymph nodes 7 days post immunization with 0.1 µg AlexaFluor 647-labeled HPV16 L1 and saponin adjuvant from C57Bl/6, MBL KO, and C3 KO mice and percent HPV16 L1 signal found within follicles. Error bars indicate SEM; points represent average values between paired draining lymph nodes from one animal; p***<0.001; p****<0.0001, ns=not significant by one-way ANOVA followed by Tukey post hoc test. **e** C57Bl/6 or C3 KO mice (n=5/group) were immunized with 0.1 µg HPV16 L1 and saponin adjuvant. Absolute counts of germinal center B cells ($B220^+GL7^+CD4^-CD38^{low}$) and antigen-specific germinal center B cells ($B220^+GL7^+HPV16\ L1^+CD4^-CD38^{low}$) were obtained after 12 days. Error bars indicate SEM, p*<0.05 by Mann-Whitney test. **f** Analyses of normalized total HBsAg signal per Z plane of cleared lymph nodes 7 days post immunization with 5 µg AlexaFluor 647-labeled HBsAg and saponin adjuvant from C57Bl/6, MBL KO, µIgM KO, and C3 KO mice and percent HBsAg signal found within follicles. Error bars indicate SEM; points represent average values between paired draining lymph nodes from one animal; p*<0.05; p**<0.01; p***<0.001; p****<0.0001, ns=not significant by one-way ANOVA followed by Tukey post hoc test. **g** C57Bl/6 or MBL KO mice (n=5/group) were immunized with 5 µg HBsAg and saponin adjuvant. Absolute counts of germinal center B cells ($B220^+GL7^+CD4^-CD38^{low}$) and antigen-specific germinal center B cells ($B220^+GL7^+HBsAg^+CD4^-CD38^{low}$) were obtained after 12 days. Error bars indicate SEM, ns=not significant by Mann-Whitney test.

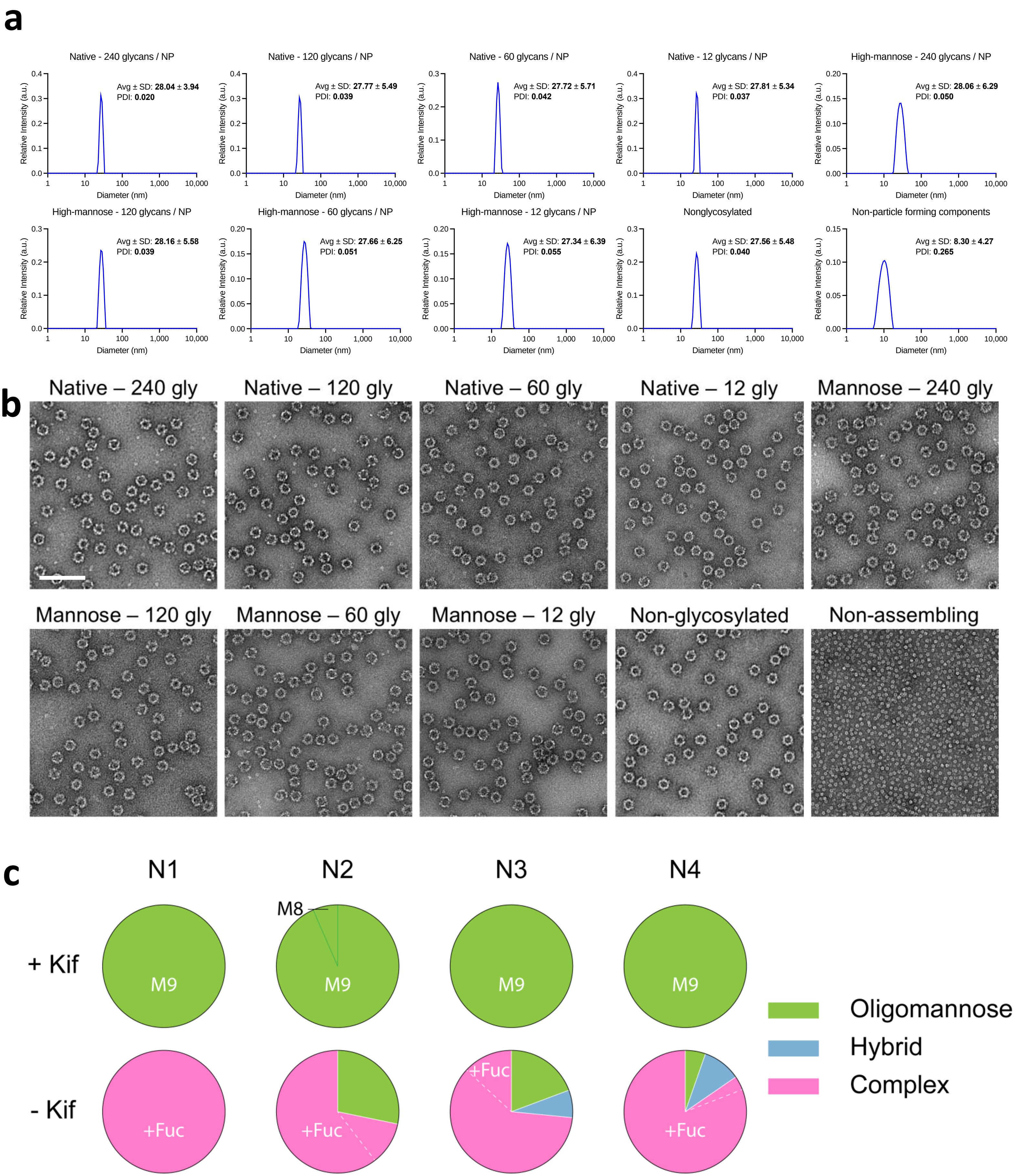

a
Native - 240 glycans / NP
Avg ± SD: 28.04 ± 3.94
PDI: 0.020
Native - 120 glycans / NP
Avg ± SD: 27.77 ± 5.49
PDI: 0.039
Native - 60 glycans / NP
Avg ± SD: 27.72 ± 5.71
PDI: 0.042
Native - 12 glycans / NP
Avg ± SD: 27.81 ± 5.34
PDI: 0.037
High-mannose - 240 glycans / NP
Avg ± SD: 28.06 ± 6.29
PDI: 0.050
High-mannose - 120 glycans / NP
Avg ± SD: 28.16 ± 5.58
PDI: 0.039
High-mannose - 60 glycans / NP
Avg ± SD: 27.66 ± 6.25
PDI: 0.051
High-mannose - 12 glycans / NP
Avg ± SD: 27.34 ± 6.39
PDI: 0.055
Nonglycosylated
Avg ± SD: 27.56 ± 5.48
PDI: 0.040
Non-particle forming components
Avg ± SD: 8.30 ± 4.27
PDI: 0.265
Relative Intensity (a.u.)
Diameter (nm)
b
Native – 240 gly
Native – 120 gly
Native – 60 gly
Native – 12 gly
Mannose – 240 gly
Mannose – 120 gly
Mannose – 60 gly
Mannose – 12 gly
Non-glycosylated
Non-assembling
c
N1
N2
N3
N4
+ Kif
- Kif
M8
M9
+Fuc
Oligomannose
Hybrid
Complex

Figure S4. Characterization of glycosylated I53-50 nanoparticles. Related to Figure 6. a Hydrodynamic diameter of glycosylated and non-glycosylated I53-50 nanoparticles and non-assembling control as determined by dynamic light scattering. **b** Negative-stain TEM of glycosylated and non-glycosylated I53-50 nanoparticles and non-assembling control (scale bar, 100 nm). **c** Pie charts of glycan compositions at four N-linked glycosylation sites on I53-50A with and without kifunensine (Kif) treatment (M9: Mannose-9; M8: Mannose-8; +Fuc: Fucosylated glycans).

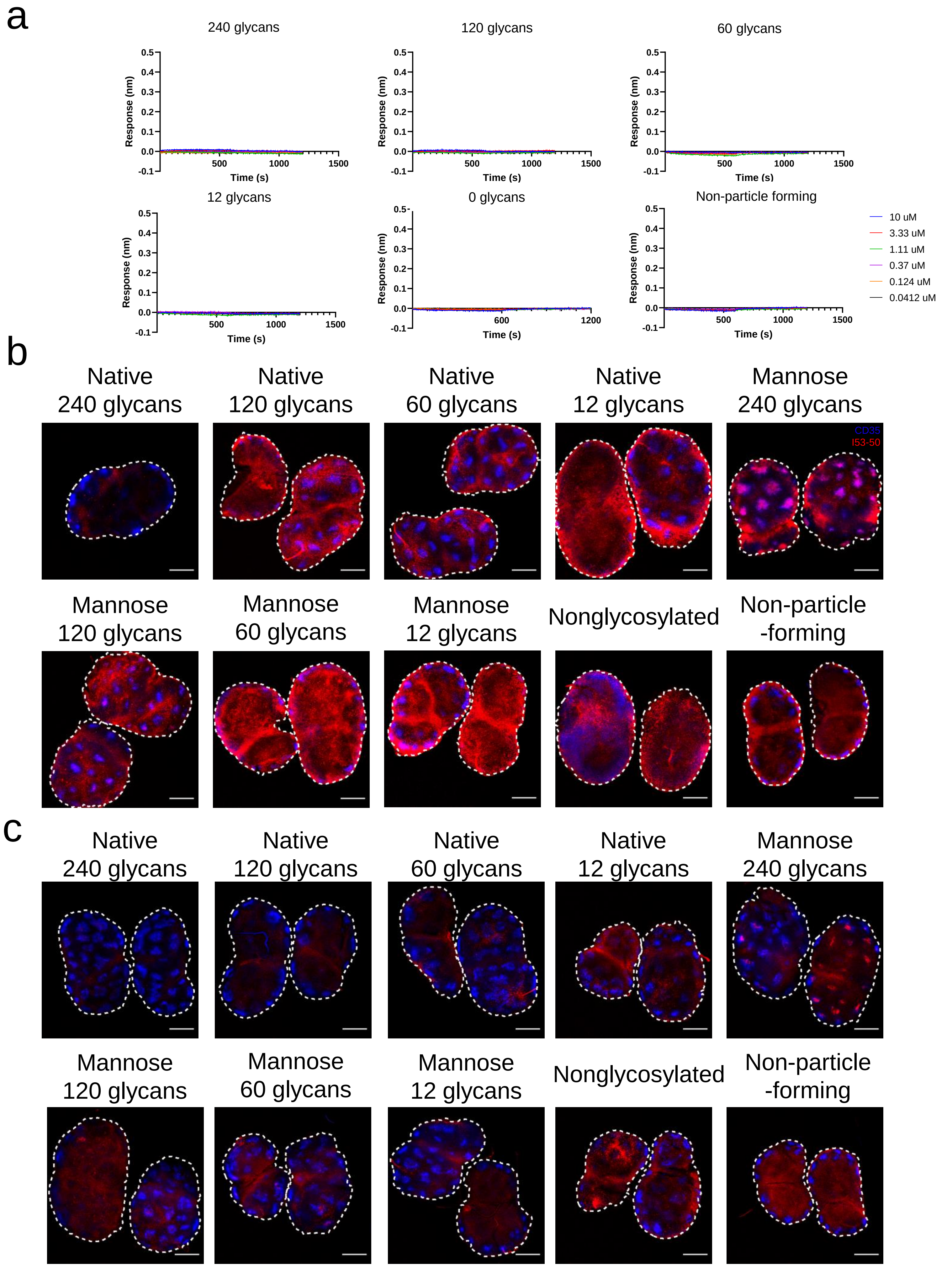
a
240 glycans
120 glycans
60 glycans
12 glycans
0 glycans
Non-particle forming
Response (nm)
Time (s)
10 uM
3.33 uM
1.11 uM
0.37 uM
0.124 uM
0.0412 uM
b
Native 240 glycans
Native 120 glycans
Native 60 glycans
Native 12 glycans
Mannose 240 glycans
CD35
I53-50
Mannose 120 glycans
Mannose 60 glycans
Mannose 12 glycans
Nonglycosylated
Non-particle -forming
c
Native 240 glycans
Native 120 glycans
Native 60 glycans
Native 12 glycans
Mannose 240 glycans
Mannose 120 glycans
Mannose 60 glycans
Mannose 12 glycans
Nonglycosylated
Non-particle -forming

Figure S5. *In vitro* MBL binding and lymph node localization of I53-50 nanoparticles. Related to Figure 6. a BLI analysis of differentially glycosylated I53-50 nanoparticles binding bearing native, complex glycans to immobilized recombinant murine MBL2 as a function of I53-50 nanoparticle concentration. **b-c** C57Bl/6 mice (n=5/group) were immunized with 5 µg AF647-labeled I53-50 glycan variants and saponin adjuvant. Shown are average intensity Z projections through 360 µm of cleared draining lymph nodes harvested on days 3 (**b**) and 7 (**c**) (blue, CD35; red, I53-50; scale bars denote 500 µm).

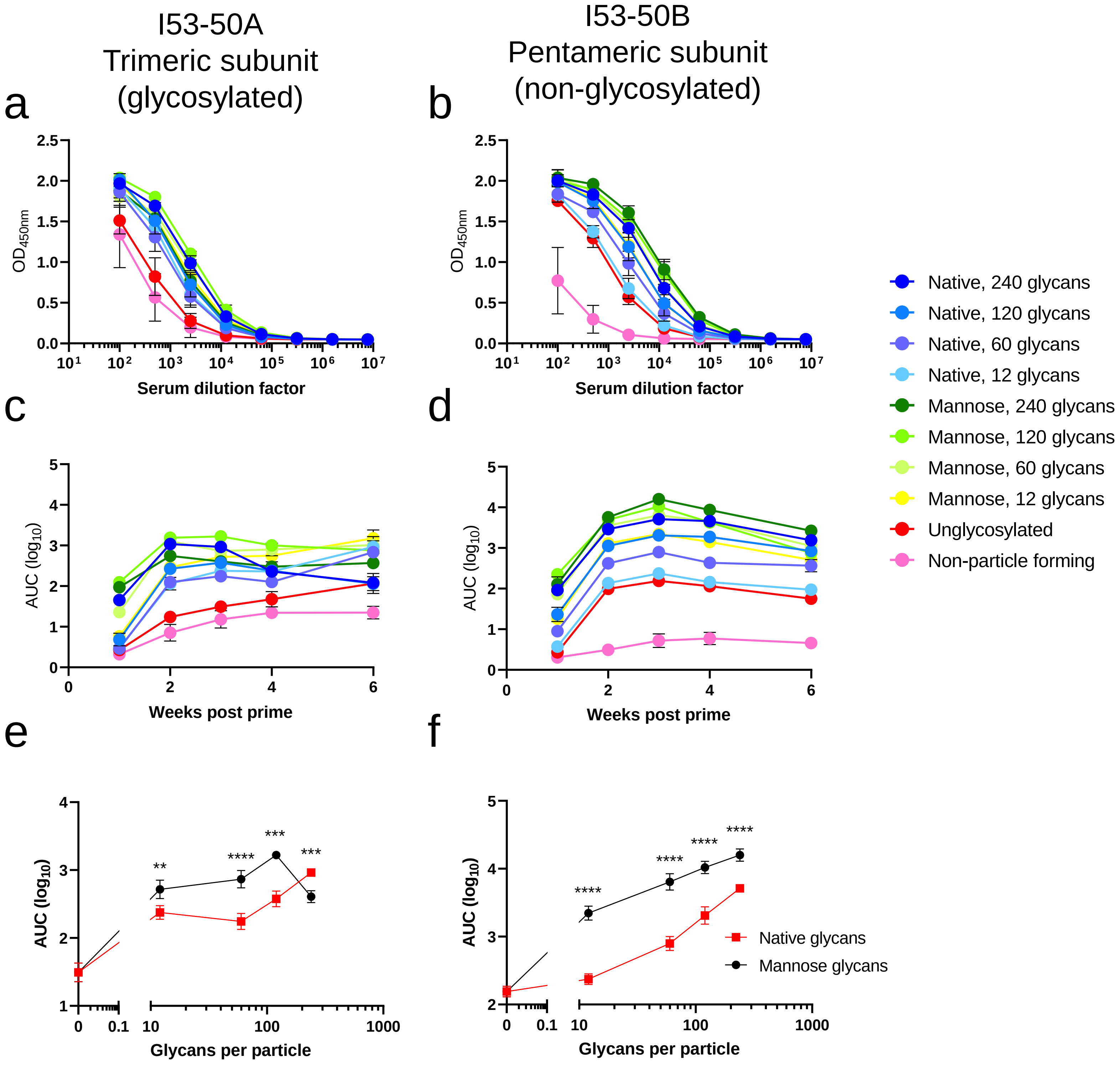

Figure S6. Antibody responses following I53-50 immunization. Related to Figure 6. C57Bl/6 mice (n=5/group) were immunized with 5 µg I53-50 glycan variants and saponin adjuvant. **a-b** Shown are raw ELISA titration curves from three weeks post immunization against I53-50A trimer (**a**) and I53-50B pentamer (**b**) particle subunits. **c-d** AUC measurements of raw ELISA titration curves through six weeks post immunization against I53-50A trimer (**c**) and I53-50B pentamer (**d**) particle subunits. **e-f** Relationship between glycans per particle and AUC of raw ELISA titration curves at three weeks post immunization against I53-50A trimer (**e**) and I53-50B pentamer (**f**) particle subunits. Error bars indicate SEM; p**<0.01; p***<0.001; p****<0.0001 by Mann-Whitney test.
