# Supplementary material for: Mannose-binding lectin and complement mediate follicular localization and enhanced immunogenicity of diverse protein nanoparticle immunogens
Source: Cell Rep. Author manuscript; Available in PMC 2022 Feb 1. (PMC8805147; doi:10.1016/j.celrep.2021.110217)
Supplement: 2 [file NIHMS1771086-supplement-2.pdf]

Cell Reports

Article

Mannose-binding lectin and complement mediate follicular localization and enhanced immunogenicity of diverse protein nanoparticle immunogens

Graphical abstract

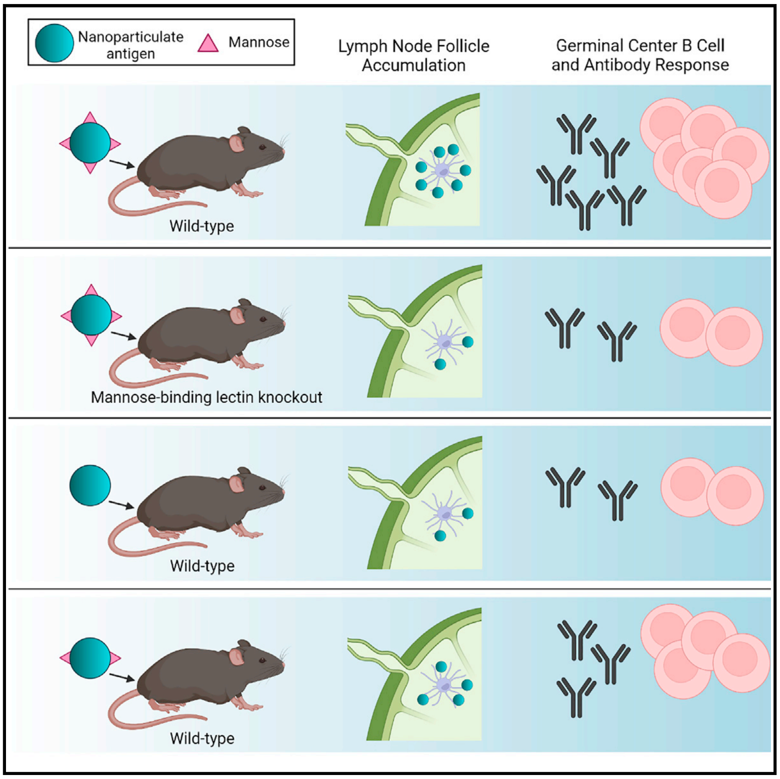

Highlights

- Diverse mannosylated nanoparticulate antigens accumulate in lymph node follicles
- Trafficking is most often dependent on complement recognition and surface glycans
- Trafficking is associated with enhanced antigen-specific immune responses
- Titration of surface mannose reveals glycan density-dependent trafficking patterns

Authors

Benjamin J. Read, Lori Won, John C. Kraft, ..., Daniel Lingwood, Neil P. King, Darrell J. Irvine

Correspondence

djirvine@mit.edu

In brief

Read et al. find that, for a diverse selection of nanoparticle antigens, lectin and complement binding of glycans on the particle surface can lead to nanoparticle accumulation within lymph nodes. This trafficking behavior correlates with enhanced immune responses and can be modulated through modifications of the displayed glycans.

Read et al., 2022, Cell Reports *38*, 110217
January 11, 2022 © 2021 The Author(s).
https://doi.org/10.1016/j.celrep.2021.110217

CellPress

Cell Reports

CellPress
OPEN ACCESS

Article

Mannose-binding lectin and complement mediate follicular localization and enhanced immunogenicity of diverse protein nanoparticle immunogens

Benjamin J. Read,[1,2] Lori Won,[1,3] John C. Kraft,[4,5] Isaac Sappington,[4,5] Aereas Aung,[1] Shengwei Wu,[1] Julia Bals,[6] Chengbo Chen,[7,8] Kelly K. Lee,[7,8] Daniel Lingwood,[6] Neil P. King,[4,5] and Darrell J. Irvine[1,3,6,9,10,11,12,*]

[1]Koch Institute for Integrative Cancer Research, Massachusetts Institute of Technology, Cambridge, MA 02139, USA
[2]Health Sciences and Technology, Harvard University and Massachusetts Institute of Technology, Cambridge, MA 02139, USA
[3]Department of Materials Science and Engineering, Massachusetts Institute of Technology, Cambridge, MA 02139, USA
[4]Department of Biochemistry, University of Washington, Seattle, WA 98195, USA
[5]Institute for Protein Design, University of Washington, Seattle, WA 98195, USA
[6]The Ragon Institute of Massachusetts General Hospital, Massachusetts Institute of Technology, Harvard University, Cambridge, MA 02139, USA
[7]Department of Medicinal Chemistry, University of Washington, Seattle, WA 98195, USA
[8]Biological Physics Structure and Design Program, University of Washington, Seattle, WA 98195, USA
[9]Consortium for HIV/AIDS Vaccine Development, The Scripps Research Institute, La Jolla, CA 92037, USA
[10]Department of Biological Engineering, Massachusetts Institute of Technology, Cambridge, MA 02139, USA
[11]Howard Hughes Medical Institute, Chevy Chase, MD 20815, USA
[12]Lead contact
*Correspondence: djirvine@mit.edu
https://doi.org/10.1016/j.celrep.2021.110217

SUMMARY

Nanoparticle (NP) vaccine formulations promote immune responses through multiple mechanisms. We recently reported that mannose-binding lectin (MBL) triggers trafficking of glycosylated HIV Env-immunogen NPs to lymph node follicles. Here, we investigate effects of MBL and complement on NP forms of HIV and other viral antigens. MBL recognition of oligomannose on gp120 nanoparticles significantly increases antigen accumulation in lymph nodes and antigen-specific germinal center (GC) responses. MBL and complement also mediate follicular trafficking and enhance GC responses to influenza, HBV, and HPV particulate antigens. Using model protein nanoparticles bearing titrated levels of glycosylation, we determine that mannose patches at a minimal density of 2.1×10^{-3} mannose patches/nm^2 are required to trigger follicular targeting, which increases with increasing glycan density up to at least $\sim 8.2 \times 10^{-3}$ patches/nm^2. Thus, innate immune recognition of glycans has a significant impact on humoral immunity, and these findings provide a framework for engineering glycan recognition to optimize vaccine efficacy.

INTRODUCTION

The use of nanoparticles displaying many copies of an antigen on their surface has been a very successful strategy for enhancing humoral responses to vaccine candidates. Nanoparticle immunogens exhibit a number of features promoting protective immunity, including multivalent antigen display, efficient lymphatic trafficking, and the potential for improved antigen stability compared with monomeric antigen formulations (Irvine and Read, 2020; Pan and Cui, 2020; Singh, 2021; Stephens and Varga, 2020; Wibowo et al., 2020). Licensed vaccines against human papillomavirus (Naud et al., 2014) and hepatitis B virus (Jackson et al., 2007) have established that nanoparticle vaccines can be safe and highly effective, and an increasing number of clinical trials are evaluating nanoparticle vaccines in which a heterologous antigen is presented on a nanoparticle scaffold, including SARS-CoV-2 (Fries et al., 2019; Keech et al., 2020; Langley et al., 2020; Madhi et al., 2021).

Despite the many potential benefits of formulating vaccine antigens as nanoparticles, key questions remain regarding fundamental design criteria to maximize nanoparticle immunogenicity. One important factor is localization of antigens in lymphoid tissues following immunization. Numerous preclinical studies have found that nanoparticles of a variety of compositions drain efficiently through the lymph, but upon reaching secondary lymphoid tissues remain primarily localized within the subcapsular sinus and the medulla or are seemingly excluded from B cell follicles (Katagiri et al., 2019; Manolova et al., 2008; Moon et al., 2012; Mueller et al., 2015; Reddy et al., 2006; Shukla et al., 2017; Wang et al., 2019). Since interactions with B cells within follicles are essential for the development of high-affinity antibodies, localization of antigen within these regions has the potential to drive more robust humoral responses. In antigen-experienced

© 2021 The Author(s).
This is an open access article under the CC BY license (http://creativecommons.org/licenses/by/4.0/).

animals, parenteral injection of antigens leads to rapid immune complex (IC) formation, complement deposition on ICs, and subsequent complement receptor-mediated trafficking of antigen and immunoglobulin to follicular dendritic cells (FDCs) (Heinen et al., 1986; Phan et al., 2009). However, this FDC trafficking pathway is not generally present during a primary immunization. Immunization with pre-formed immune complexes (Chen et al., 2016; Diamos et al., 2019; Gach et al., 2019; Martin et al., 2020) or fusion of antigens with multiple copies of complement subunits (Bergmann-Leitner et al., 2007; Dempsey et al., 1996; Movsesyan et al., 2008; Pompa-Mera et al., 2014) have the capacity to trigger similar targeting of immunogens to FDCs, but these strategies present their own complexities in implementation.

We recently reported that glycosylated HIV-immunogen-bearing nanoparticles accumulate within follicles in a process mediated by mannose-binding lectin (MBL), which triggers complement deposition on the nanoparticle surface, transport to FDCs, and enhanced germinal center (GC) and serum antibody responses relative to non-accumulating nanoparticles (Tokatlian et al., 2018). Here, we further explored this trafficking mechanism to gain insight into how broadly this pathway operates in response to diverse vaccine antigens, and to further elucidate design rules for engaging this pathway in future nanoparticle vaccines. As a clinically relevant nanoparticulate antigen, we employed an HIV immunogen, the germline-targeting engineered outer domain (eOD-GT8, herein referred to as eOD) displayed on a self-assembling lumazine synthase backbone that forms a nanoparticle (NP) consisting of 60 identical subunits (eOD-60mer) (Jardine et al., 2013, 2016, 2015). This NP immunogen was recently tested in a phase I clinical trial and was demonstrated to be highly effective in triggering activation of VRC01-class B cells as a first step toward a vaccine capable of eliciting broadly neutralizing antibodies against HIV targeting the CD4 binding site (Diemert and McElrath, 2015). We previously showed this NP immunogen exhibits robust MBL-dependent trafficking to FDCs following immunization (Tokatlian et al., 2018). This MBL trafficking pathway also depends on MBL-mediated activation of complement (as summarized schematically in Figure S1). Here, we further characterized the nature of this trafficking, its impact on GC responses within draining lymph nodes, and the glycan requirements for MBL binding to eOD-60mer. We then assessed the trafficking patterns of other clinically relevant NP antigens and their ability to engage with MBL to ascertain the broader relevance of MBL-mediated follicular antigen accumulation. Finally, we created a panel of model protein NPs bearing titrated levels of complex or high-mannose glycans to extract insights into design rules for engineering MBL-dependent follicular homing into vaccines more generally.

RESULTS

Protein nanoparticles of eOD-GT8 antigen concentrate in B cell follicles in an MBL-dependent manner

We previously analyzed the distribution of eOD-GT8 monomer and 60mer immunogens in lymph nodes following immunization by imaging cleared whole lymph node tissues, and we observed selective MBL-dependent accumulation of eOD-60mer NPs in B cell follicles, while eOD monomer was present at very low levels dispersed through the lymph node parenchyma (Tokatlian et al., 2018). Similar observations were made by imaging traditional histological tissue sections. However, to rule out the possibility that these findings were biased by selective extraction of monomer or extra-follicular antigen from the tissues during tissue clearing or histological sample preparation, we repeated this analysis using two approaches designed to avoid any potential antigen loss. First, wild-type (WT) or MBL KO mice were immunized with far-red dye-labeled eOD monomer or eOD-60mer administered subcutaneously with saponin adjuvant, followed by excision of draining inguinal lymph nodes (iLNs) and immediate whole-tissue fluorescence imaging to measure antigen accumulation and persistence in the tissues. In WT mice, eOD-60mer showed high levels of accumulation by day 3 post injection, and antigen persisted through at least 1 week. By contrast, the 60mer immunogen showed significantly lower accumulation in iLNs of MBL KO mice, which decayed to near baseline by day 7 (Figure 1A). Next, we repeated these experiments with both eOD monomer and eOD-60mer, but instead of whole-tissue imaging, lymph nodes were excised, flash frozen, and sectioned using a cryomicrotome to image minimally manipulated tissue sections. Confocal imaging of these samples revealed that in all treatment groups, eOD antigen was present within the subcapsular sinus 1 day following immunization, though lymph nodes from mice immunized with eOD monomer had less detectable antigen than nodes from either eOD-60mer group. Mice immunized with eOD-60mer had significant levels of antigen detectable in both the subcapsular sinus and the medulla 1 day after immunization. Interestingly, however, a larger proportion of eOD-60mer was found within the medulla in nodes from MBL KO mice than from WT mice, suggesting differing antigen trafficking and capture between the two mouse strains. Three days post immunization, significant levels of antigen were only detected in nodes from WT mice immunized with eOD-60mer, and the majority of this antigen was found within B cell follicles (Figures 1B and S2A). Further, a majority of follicles from WT mice immunized with eOD-60mer contained antigen deposits, a phenomenon that was not observed in MBL KO mice (Figure 1C). Thus, using experiments designed to avoid potential extraction of antigen from the tissues, the NP form of the eOD-GT8 immunogen was found to show enhanced accumulation in lymph nodes over the monomeric form of the antigen, with antigen accumulation and follicular trafficking dependent upon the innate immune protein MBL.

MBL and complement recognition amplify antigen-specific germinal center responses and serum antibody titers following eOD-GT8 nanoparticle immunization

We previously showed that MBL KO animals exhibited reduced total GC responses and lower serum antibody responses to immunization with eOD-GT8 60mer particles (Tokatlian et al., 2018). Motivated by these findings, we sought to examine in further detail the role of MBL and complement in the humoral immune response to these NP immunogens. We first assessed the impact of MBL deficiency on total versus antigen-specific B cell responses, using fluorescent eOD-60mer probes to identify cognate B cells. Total antigen-specific B cells were reduced by

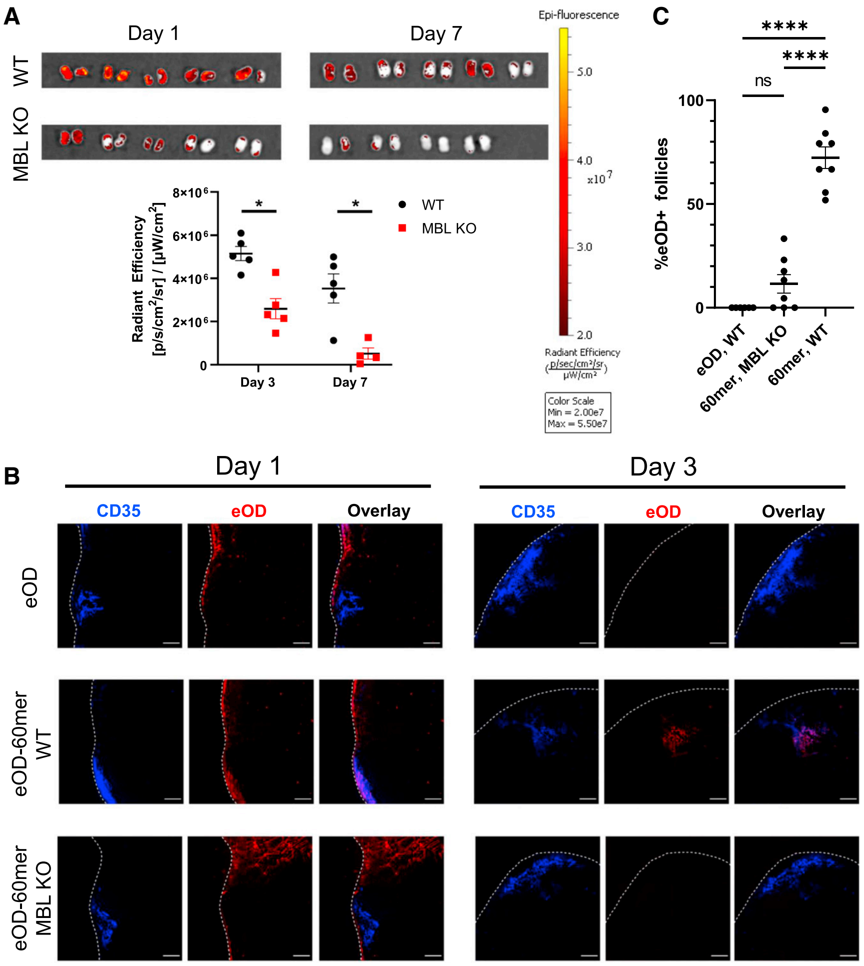

Figure 1. eOD-GT8 60mer nanoparticles accumulate in lymph nodes and localize to the FDC network of follicles in an MBL-dependent manner

(A) C57Bl/6 or MBL KO mice (n = 4–5/group) were immunized with 2 μg eOD equivalent AlexaFluor 647-labeled eOD-GT8 60mer and saponin adjuvant. Three or seven days post immunization, draining inguinal lymph nodes were excised for whole-tissue fluorescence imaging. Error bars indicate SEM; *, $p < 0.05$ by Mann-Whitney test.

(B) C57Bl/6 or MBL KO mice (n = 5/group) were immunized with 2 μg eOD equivalent AlexaFluor 647-labeled eOD-GT8 60mer or eOD monomer and saponin adjuvant. One or three days post immunization, lymph nodes were snap-frozen and cryosectioned into six sections each for confocal imaging. Blue, CD35; red, eOD-GT8; scale bars denote 100 μm.

(C) C57Bl/6 or MBL KO mice (n = 4/group) were immunized with 2 μg AF647-labeled eOD or 2 μg eOD equivalent AF647-labeled eOD-GT8 60mer and saponin adjuvant. Draining lymph nodes were harvested on day 7 and cleared for confocal imaging. Shown are the percentage of antigen-positive follicles among all follicles within each individual draining lymph node. Error bars indicate SEM; ****, $p < 0.0001$; ns, not significant by Mann-Whitney test.

~53% in MBL KO animals relative to WT mice, while total GC B cells were reduced by ~63% and total antigen-specific GC B cells were reduced by ~58% (Figures 2A–2F). Interestingly, the MFI of eOD-GT8 staining among antigen-specific GC B cells did not differ between WT and MBL KO mice (Figure 2G), while total surface IgG expression by these cells was similar (Figure S2B), which may indicate that those antigen-specific B cells that do develop in MBL KO animals mature to reach affinities for antigen comparable to B cells from WT mice.

MBL binding to eOD-60mer leads to complement deposition on the NPs through the lectin pathway of complement activation (Tokatlian et al., 2018). We previously found that in addition to MBL, eOD-60mer localization to follicles is dependent on the presence of an intact complement pathway and complement receptors 1/2. Consistent with these findings, complement component C3, a critical molecule for all complement activation pathways, was found to deposit on eOD-60mer at substantially reduced levels *in vitro* when the NP was incubated in serum from MBL KO mice versus serum from WT mice (Figure S2C). We next examined the relative importance of complement versus MBL in the output serum antibody response. As we previously reported (Tokatlian et al., 2018), immunization of MBL KO animals with eOD-60mer elicited ~4-fold lower antigen-specific IgG titers in the serum, and this difference was maintained over time. However, humoral responses were more strikingly completely ablated in C3 KO mice lacking a functioning complement system and in CR1/2 KO mice lacking complement receptors on B cells and FDCs (Figure 2H). Hence, although MBL acts in part through complement activation, the complement pathway is more critical for optimal B cell priming in response to eOD-60mer particles.

Mannose glycans on eOD-GT8 particles are required for MBL binding and follicle accumulation

While MBL is known to preferentially bind to certain classes of glycans (Takahashi et al., 2006; Turner, 2003), it remained unclear which glycans mediate MBL recognition and subsequent trafficking of glycosylated NPs *in vivo*. To investigate this question, we first evaluated what type of glycosylation was required for MBL binding *in vitro* by producing six differently glycosylated forms of eOD-GT8 60mer: eOD-GT8 NPs were expressed in Expi293 cells in the presence of kifunensine to create particles bearing only high-mannose glycosylation (HM 60mer), or in the absence of kifunensine (complex 60mer) to obtain particles with ~75% complex glycans and only ~25% high-mannose glycans (Tokatlian et al., 2018). These two types of particle were further treated with endoglycosidase H (Endo H) to remove high-mannose moieties or PNGase F to remove all glycans. Both HM 60mer bearing only high-mannose glycans and complex 60mer bearing predominantly complex glycans bound to immobilized MBL in a concentration-dependent manner as

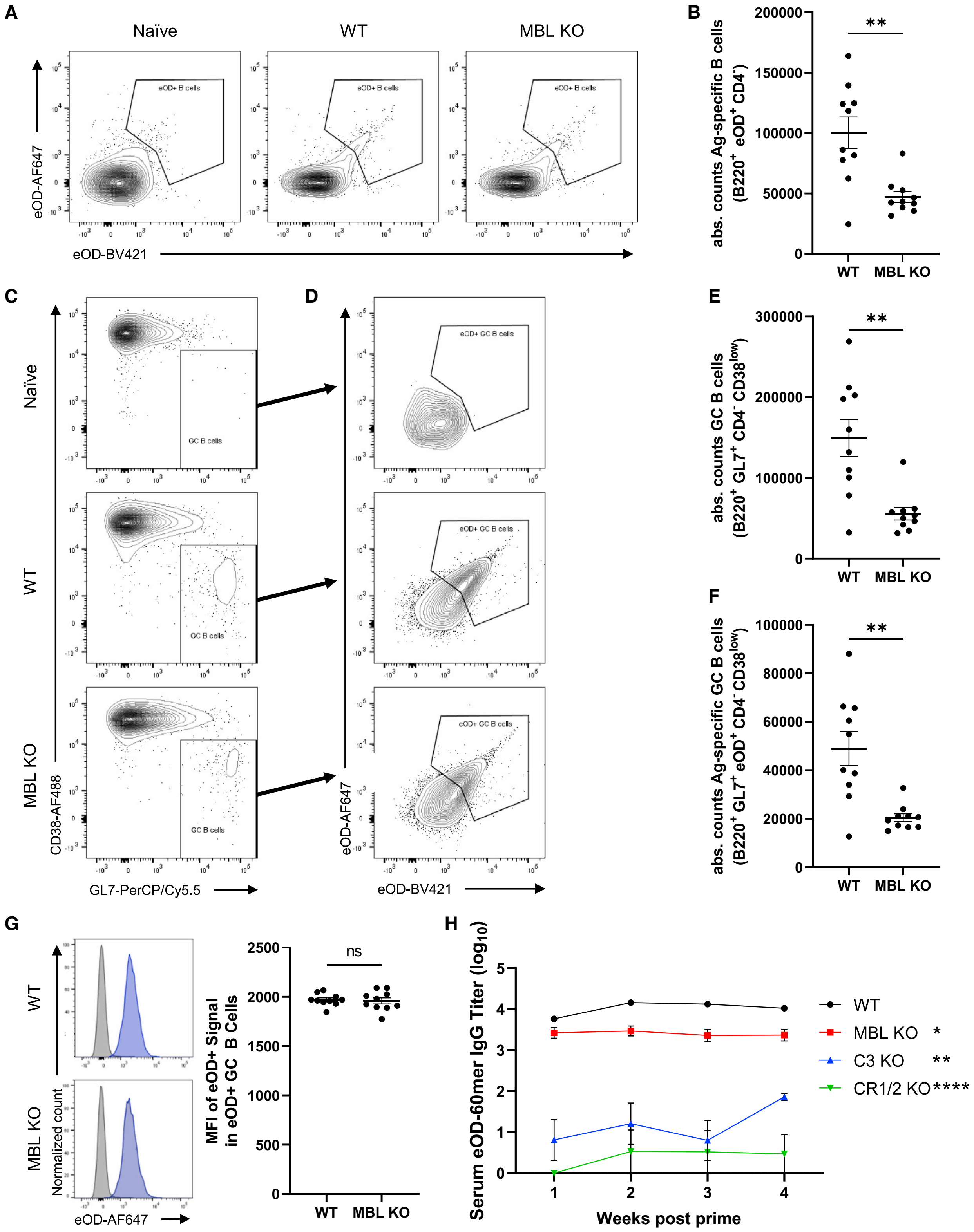

(legend on next page)

measured by biolayer interferometry (BLI), but binding was greater for the 60mer bearing only oligomannose sugars (Figure 3A). As expected, treatment with PNGase F similarly ablated all binding of MBL to both HM and complex eOD-GT8 60mer particles *in vitro*. Notably, treatment of the NPs with Endo H ablated binding for both high-mannose- and complex glycan-bearing particles as well, indicating that MBL requires mannose for recognition of the NP immunogen (Figure 3A). Complex but not HM 60mer was found to bear exposed sialic acid residues, but selective removal of sialic acids via neuraminidase treatment did not alter MBL binding (Figures S2D and S2E).

To assess the biological impact of these distinct MBL binding patterns, we imaged the localization of each of the eOD-60mer glycoforms in draining iLNs following immunization. Strikingly, while both high-mannose and complex glycan particles concentrated in follicles by 3 days post injection, Endo H- or PNGase F-treated particles lacking high-mannose glycans exhibited greatly reduced total lymph node accumulation and substantially reduced follicular accumulation (Figures 3B–3D). High-mannose and complex 60mer elicited similar eOD-specific IgG responses in WT mice as well as similarly depressed responses in MBL KO mice, correlating with their similar trafficking patterns in the lymph nodes (Figure S2F). Thus, the follicular trafficking and immunogenicity of these HIV Env NPs is linked to MBL recognition of mannose moieties on the particle surfaces.

MBL and complement mediate follicular accumulation of multiple clinically relevant nanoparticle immunogens

To understand the degree to which other clinically relevant NP antigens are affected by MBL-mediated trafficking, we evaluated the follicular localization and immune responses of several additional, non-HIV immunogen-bearing NPs. First, we investigated a self-assembling ferritin NP displaying eight copies of the influenza hemagglutinin trimer (HA-8mer), which has been previously characterized (Kanekiyo et al., 2013; Weaver et al., 2016) and is similar to hemagglutinin-displaying NPs currently in clinical trials (Chen, 2017; Widge, 2019). In previous studies, we found that HA-8mer, which is decorated by a mix of high-mannose and complex glycans, localizes to follicles in WT mice but not in MBL KO mice (Tokatlian et al., 2018). Following immunization, total lymph node accumulation of HA-8mer was similar for WT and MBL KO mice, but the particles accumulated in follicles in WT mice versus being diffusely distributed in MBL KO animals (Figures 4A and 4B). Similar to our findings with eOD-60mer, antibody titers against HA-8mer were substantially reduced, by ~10-fold, in MBL KO versus WT mice (Figure 4C). An analysis of GCs showed that WT mice had significantly higher levels of total GC B cells as well as antigen-specific GC B cells (Figures 4D and 4E). Also similar to our findings with eOD-60mer, there was no significant difference between the mean fluorescent intensities of the HA-8mer stain within the antigen-specific GC B cell populations between groups at day 12 (Figure 4F).

Next, we examined the capacity of two self-assembling NP antigens currently used for human vaccinations, human papillomavirus 16 L1 virus-like particles (HPV16 L1) and hepatitis B virus surface antigen virus-like particles (HBsAg), to engage with MBL trafficking. Dynamic light scattering confirmed these immunogens formed particles of the expected sizes (Figures S3A and S3B). By BLI analysis, HPV16 L1 exhibited concentration-dependent binding to immobilized recombinant murine MBL, which was greatly reduced following PNGase F treatment (Figure 5A). By contrast, MBL showed no binding to HBsAg even at high particle concentrations (Figure 5B). Imaging of fluorescently labeled HPV16 L1 in lymph nodes 7 days post immunization revealed follicular accumulation of HPV particles in WT but not in MBL KO animals (Figure 5C) or in C3 KO animals (Figures S3C and S3D). Distinct from the other NPs evaluated thus far, immunization with HPV16 L1 elicited serum antibody titers that were not statistically different in WT versus MBL KO mice, though in C3 KO mice it was drastically reduced (Figure 5D). However, both total and antigen-specific GC B cells were reduced by ~50% in MBL KO animals compared with WT mice (Figure 5E) and were almost completely ablated in C3 KO animals (Figure S3E). Interestingly, fluorescent HBsAg particles exhibited essentially identical follicular accumulation in both WT and MBL KO animals at 7 days post immunization (Figures 5F and S3F). This prompted us to test whether these particles might be trafficked via complement activation through the alternative pathway, or perhaps trigger complement through natural IgM (Ehrenstein and Notley, 2010; Link et al., 2012); HBsAg follicular accumulation remained present in secretory IgM-deficient (μIgM KO) animals but was lost entirely in C3 KO mice (Figures 5F and S3F), suggesting a possible role for the alternative pathway of complement activation for these NPs. WT and MBL KO mice immunized with HBsAg particles exhibited similar serum antibody responses, while C3 KO mice had significantly reduced IgG titers, though the reduction was to a lesser extent than was seen for other immunogens in C3 KO mice (Figure 5G). Immunization with HBsAg also led to substantially lower levels of total and antigen-specific GC B cells in C3 KO versus WT animals, while no difference was observed between WT and MBL KO animals (Figures 5H and S3G). In summary, in addition to HIV Env and influenza NPs, clinically relevant HPV and HBV virus-like particle vaccines exhibit MBL- or complement-dependent follicular accumulation following primary immunization;

Figure 2. MBL KO mice have reduced germinal center and antibody responses to eOD-60mer immunization

C57Bl/6 or MBL KO mice (n = 10/group) were immunized with 2 μg eOD-equivalent eOD-GT8 60mer and saponin adjuvant or PBS control.

(A–G) Flow cytometry analysis of GC B cell responses in draining inguinal lymph nodes 12 days post immunization. Shown are representative flow cytometry plots gating for antigen-specific B cells (A), absolute counts of $B220^+eOD^+CD4^-$ antigen-specific B cells (B), representative plots of GC B cells (C) and antigen-specific GC B cells (D), absolute counts of total $B220^+GL7^+CD4^-CD38^{low}$ GC B cells (E), absolute counts of total $B220^+GL7^+eOD^+CD4^-CD38^{low}$ antigen-specific GC B cells (F), and representative histograms of eOD signal MFI among all cells (gray) and antigen-specific GC B cells (blue) and the antigen-specific GC B cell eOD signal MFI from each sample (G). Error bars indicate SEM; **, p <0.01; ns, not significant by Mann-Whitney test.

(H) Serum eOD-specific IgG titers over time in mice immunized with eOD-GT8 60mer. Error bars indicate SEM; *, p <0.05; **, p <0.01; ***, p <0.0001 relative to WT by one-way ANOVA followed by Tukey post hoc test.

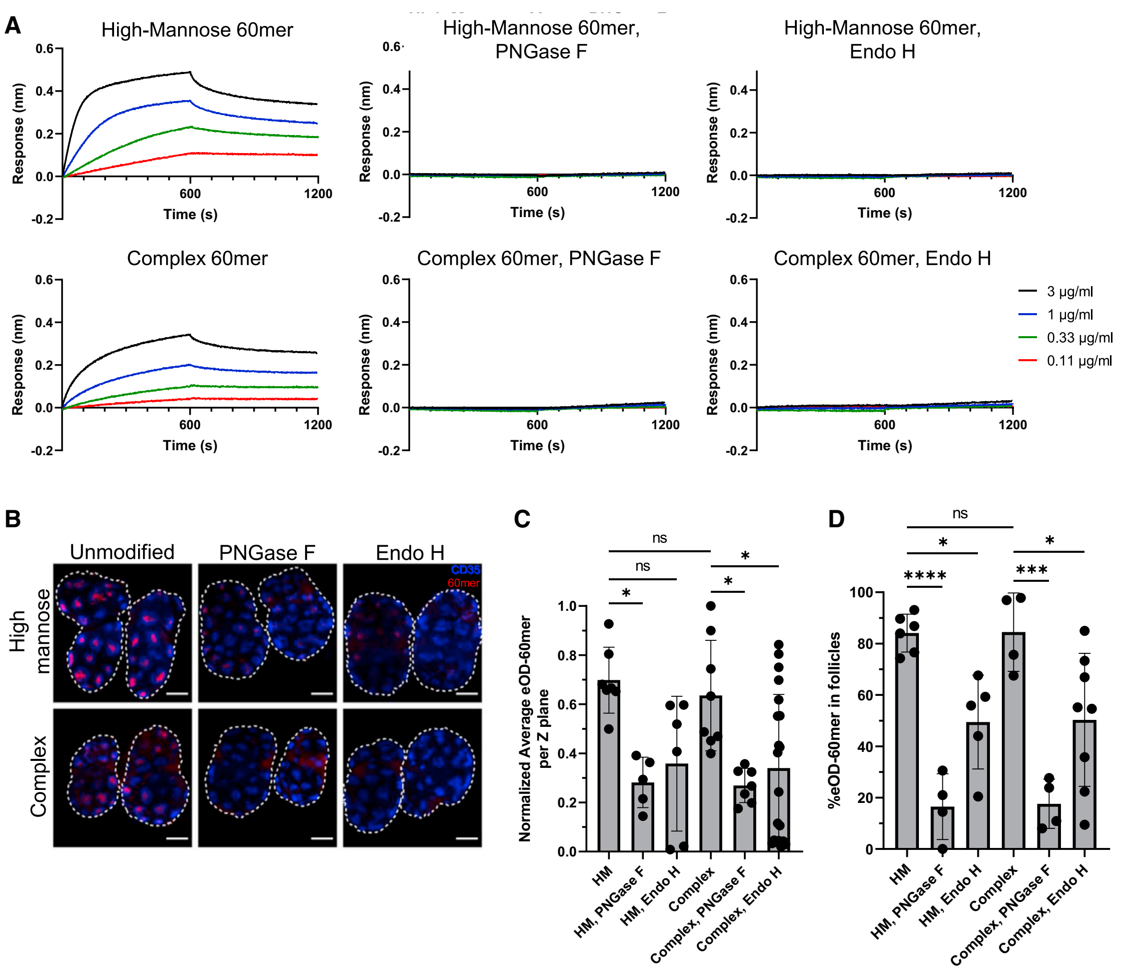

Figure 3. High-mannose glycans are required for MBL-driven follicular accumulation of eOD-GT8 nanoparticles

(A) BLI analysis of eOD-GT8 60mer glycan variants binding to immobilized recombinant murine MBL2 as a function of eOD particle concentration.

(B–D) C57Bl/6 mice (n = 5/group) were immunized with 2 μg eOD equivalent eOD-GT8 60mer glycan variants and saponin adjuvant. Shown are average intensity Z projections through 360 μm of cleared draining lymph nodes harvested on day 7 (B, blue, CD35; red, eOD-GT8 60mer; scale bars denote 500 μm), and analyses of normalized total eOD-GT8 60mer signal per Z plane of cleared lymph nodes (C) and percent eOD-60mer signal found within follicles (D). Error bars indicate SEM; points represent average values between paired draining lymph nodes from one animal; *, p <0.05; ***, p <0.001; ****, p <0.0001, ns = not significant by one-way ANOVA followed by Tukey post hoc test.

localization to FDCs uniformly correlates with enhanced GC responses, and in all but one case examined here, FDC localization correlated with increased serum antibody titers.

MBL recognition of nanoparticles of varying glycan density

Motivated by the significant impact of MBL engagement on GC responses with diverse antigens, we next sought to understand how the degree and type of glycosylation influences *in vivo* trafficking and immunogenicity of NPs. To do so, we took advantage of a designed two-component protein NP system to systematically vary glycan density and type. We selected the two-component, computationally designed, self-assembling protein nanoparticle I53-50, which forms 120-subunit NPs ~25 nm in diameter through the self-assembly of 20 trimeric I53-50A and 12 pentameric I53-50B building blocks (Figure 6A) (Bale et al., 2016). These particles have been shown to be exceptionally thermodynamically stable and have been used preclinically to scaffold several viral glycoprotein antigens, and a SARS-CoV-2 vaccine based on the I53-50 design is currently in phase III trials (Arunachalam et al., 2021; Brouwer et al., 2019, 2021; Marcandalli et al., 2019; Walls et al., 2020). In the current study, I53-50 particles with varying mean glycan densities and bearing no additional non-scaffold antigens were assembled *in vitro* by mixing glycosylated and non-glycosylated trimeric I53-50A building blocks in selected ratios together with non-glycosylated I53-50B pentamers (Figures 6A and 6B). We compared a series of particles bearing either high-mannose or complex glycans, which were assembled from trimers secreted from Expi293F cells in the presence or absence of kifunensine, respectively. All *in vitro* assembly reactions resulted in the formation of stable NPs as assessed by dynamic light scattering and negative-stain

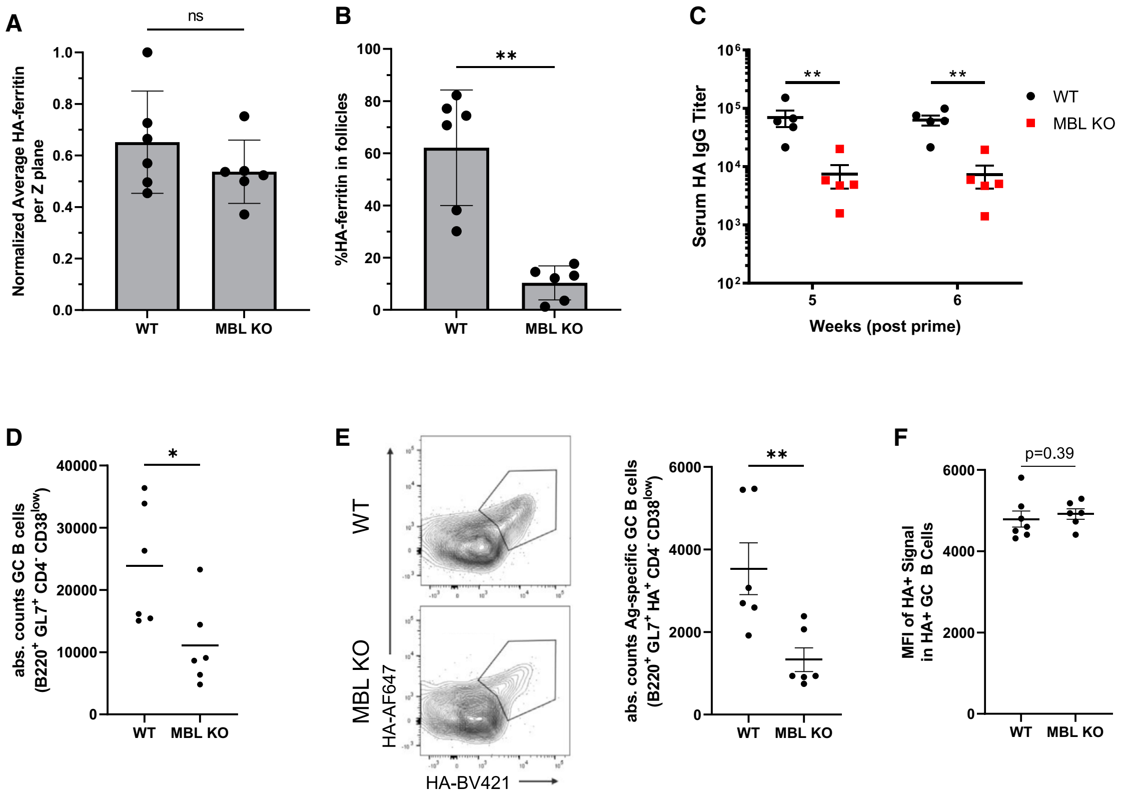

Figure 4. HA-8mer follicular accumulation and immunogenicity are dependent on MBL

(A and B) C57Bl/6 or MBL KO mice (n = 5/group) were immunized with 5 μg AlexaFluor 647-labeled influenza HA-8mer particles and saponin adjuvant. Draining lymph nodes were harvested on day 7 and cleared for confocal imaging. Shown are total HA-8mer signal per Z plane of cleared tissues (A) and percent HA-8mer signal found within follicles (B). Error bars indicate SEM; points represent average values between paired draining lymph nodes from one animal; **, p <0.01, ns = not significant by Mann-Whitney test.

(C) C57Bl/6 or MBL KO mice (n = 5/group) were immunized with 5 μg influenza HA-8mer particles and saponin adjuvant. Shown are serum hemagglutinin-specific IgG titers 5 and 6 weeks post immunization. Error bars indicate SEM; **, p <0.01 by Mann-Whitney test.

(D and F) Absolute counts of total GC B cells (D, *, p <0.05 by Mann-Whitney test) and absolute counts of antigen-specific GC B cells at day 12 (E, **, p <0.01 by Mann-Whitney test) and average MFI of antigen specificity stain among antigen-specific GC B cells (F, p = 0.39 by Mann-Whitney test).

transmission electron microscopy (Figures S4A and S4B). Analysis of glycosylation at the four N-linked glycosylation sites of the trimeric subunit by bottom-up mass spectrometry revealed that I53-50 particles prepared in the absence of kifunensine were >75% occupied by complex glycans, while those prepared with kifunensine were occupied solely by high mannose, as expected (Figure S4C). I53-50 NPs bearing only high-mannose glycans exhibited clear glycan density-dependent binding to MBL, while those bearing native, complex glycosylation were not bound by MBL at any glycan density (Figures 6C and 6D; Figure S5A). Immunization of mice with I53-50 particles displaying high-mannose glycans and formulated with saponin adjuvant led to NP accumulation within draining lymph node follicles in a clearly glycan density-dependent manner at 3 days post injection (Figures 6E and 6F). By day 7, particles with the highest glycan density continued to be detected prominently on FDCs, while the less densely glycosylated particles were beginning to be cleared. Notably, I53-50 NPs bearing native, complex glycans did not exhibit any follicular localization despite bearing the same total number of glycans (Figures S5B and S5C). This is in contrast to natively glycosylated eOD-GT8 60mer particles, which did exhibit both binding to MBL *in vitro* (Figure 3A) and efficient follicular localization (Figure 3D). The different outcome in these two cases likely reflects differences in native glycan processing of the two different protein particles, which is highly sensitive to protein structure and sequence.

Total and I53-50-specific GC responses increased for mannosylated I53-50 as the glycan density increased from 0.0049 glycans/nm^2 (12 glycans/particle) to 0.0495 glycans/nm^2 (120 glycans/particle), but we observed a drop in GC responses at the highest glycosylation level tested of 0.0989 glycans/nm^2 (240 glycans/particle) (Figure 6G). We hypothesize that this most densely glycosylated particle may have exhibited a lower GC response due to the competing effects of MBL recognition and/or FDC localization and glycan masking limiting available protein surface for antibody binding. To assess how glycosylation impacted antibody responses, we assessed serum IgG responses against the two different subunits of the particles as a function of glycan density (Figures S6A–S6D). Following a single immunization, antibody responses rapidly developed, peaking at

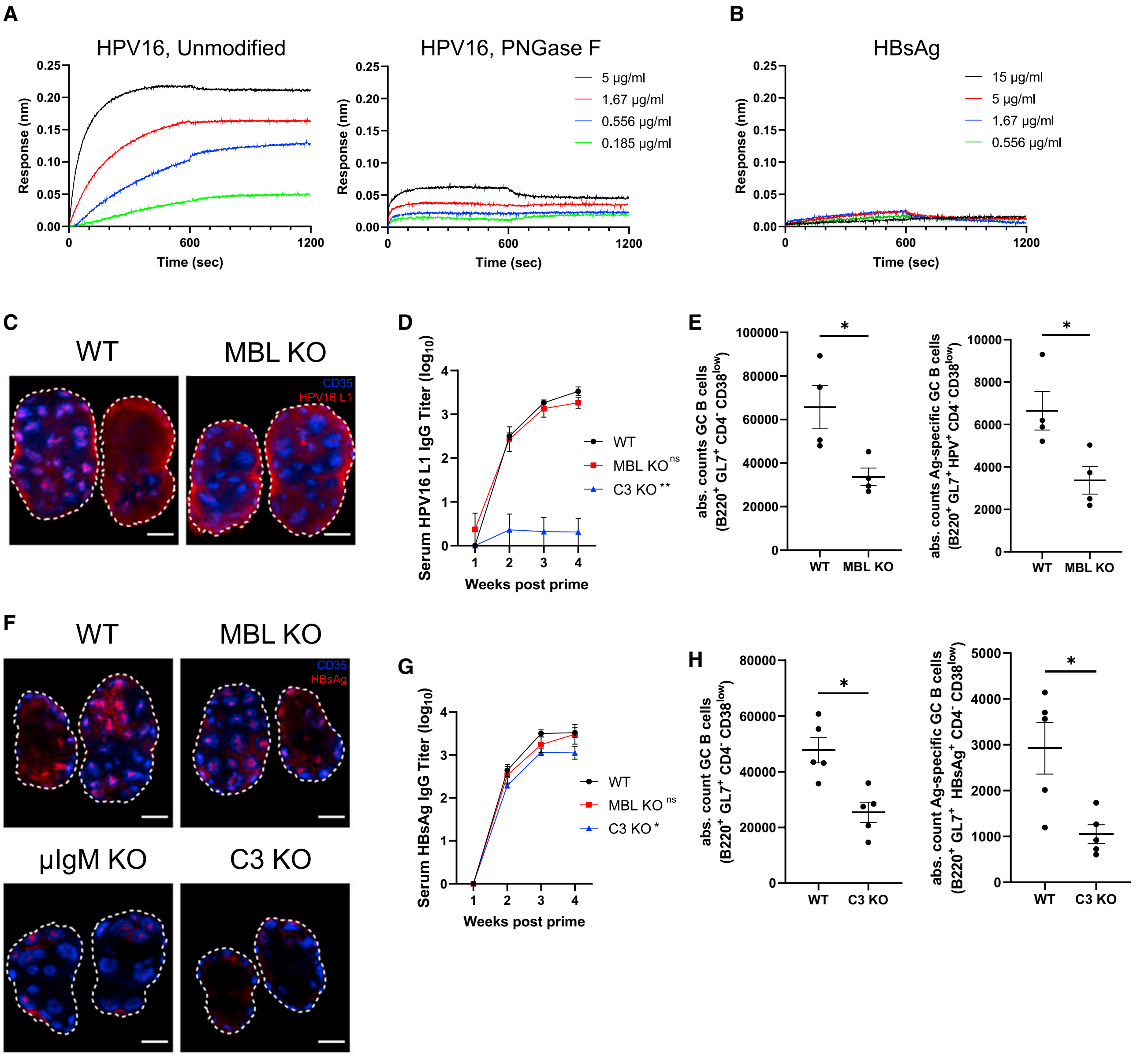

Figure 5. HPV16 L1 and HBsAg nanoparticles exhibit complement-dependent follicular accumulation and immunogenicity

(A and B) BLI binding curves of unmodified and PNGase F-treated HPV16 L1 (A) or unmodified HBsAg (B) to immobilized recombinant murine MBL2 as functions of antigen concentration.

(C) C57Bl/6 mice or MBL KO mice (n = 5/group) were immunized with 0.1 μg AlexaFluor 647-labeled HPV16 L1 and saponin adjuvant. Seven days later, lymph nodes were harvested, cleared, and imaged by confocal microscopy. Shown are average intensity Z projections through 360 μm of tissue; shown is staining for CD35 (blue) and antigen (red), scale bars denote 500 μm.

(D) Serum HPV16 L1-specific IgG titers over time in mice (n = 5/group) immunized with 0.1 μg HPV16 L1 and saponin adjuvant. Error bars indicate SEM, p = 0.92 compared with WT one-way ANOVA.

(E) Absolute counts of germinal center B cells ($B220^+GL7^+CD4^-CD38^{low}$) and antigen-specific germinal center B cells ($B220^+GL7^+HPV16\ L1^+CD4^-CD38^{low}$) from WT and MBL KO mice (n = 5/group) at day 12 following immunization with 0.1 μg HPV16 L1 and saponin adjuvant. Error bars indicate SEM; *, $p < 0.05$ by Mann-Whitney test.

(F) C57BL/6 mice or MBL KO mice (n = 5/group) were immunized with 5 μg AlexaFluor 647-labeled HBsAg and saponin adjuvant. Seven days later, lymph nodes were harvested, cleared, and imaged by confocal microscopy. Shown are average intensity Z projections through 360 μm of tissue; shown is staining for CD35 (blue) and antigen (red), scale bars denote 500 μm.

(G) Serum HBsAg-specific IgG titers over time in mice immunized with 5 μg HBsAg and saponin adjuvant. Error bars indicate SEM; *, $p < 0.05$ compared with WT by one-way ANOVA followed by Tukey post hoc test.

(H) Absolute counts of germinal center B cells ($B220^+GL7^+CD4^-CD38^{low}$) and antigen-specific germinal center B cells ($B220^+GL7^+HBsAg^+CD4^-CD38^{low}$) from WT and C3 KO mice 12 days after immunization with 5 μg HBsAg and saponin adjuvant. Error bars indicate SEM; *, $p < 0.05$ by Mann-Whitney test.

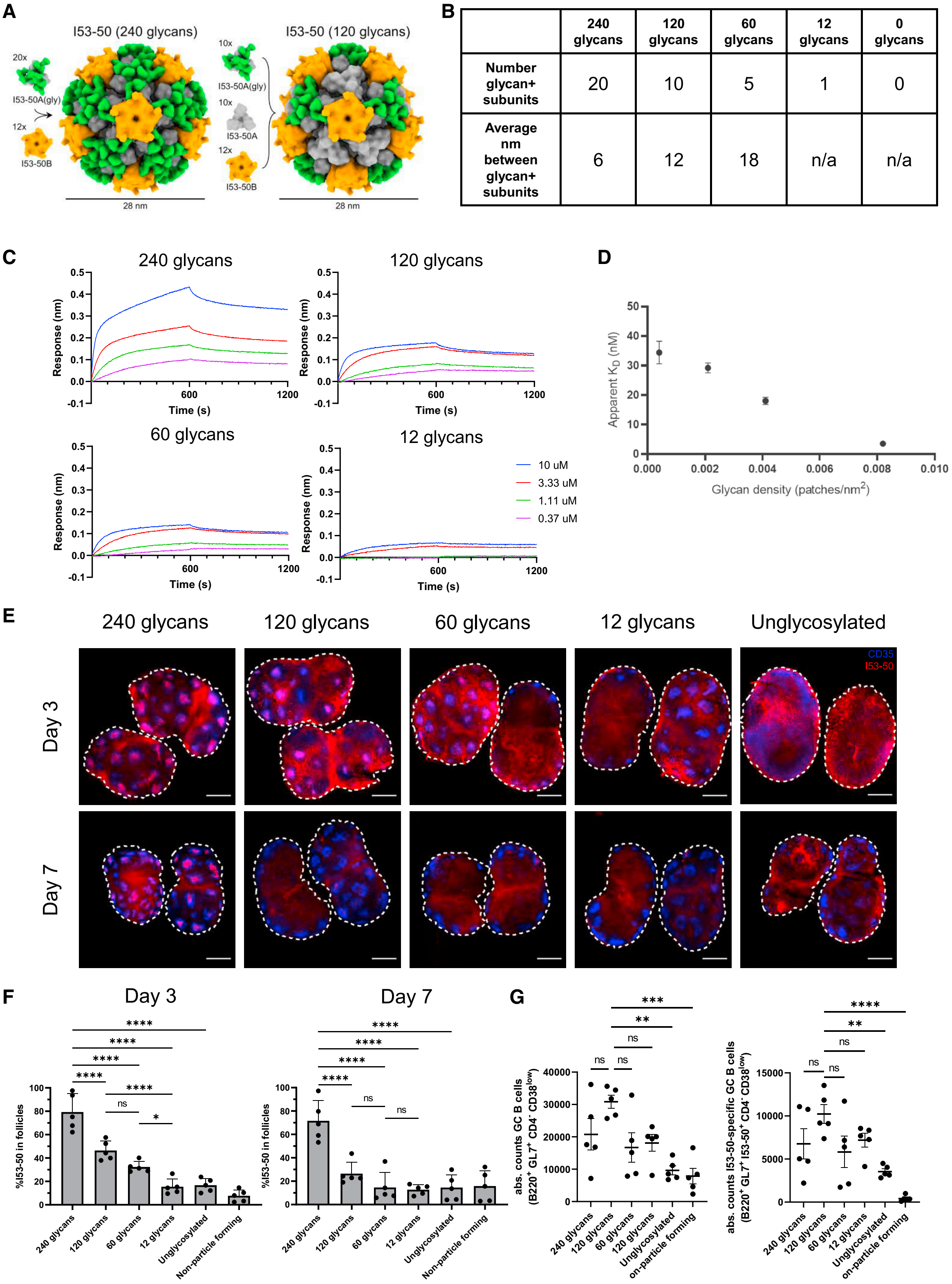

Figure 6. Differentially glycosylated nanoparticles accumulate in follicles in a mannose density-dependent manner

(A) Design models of glycosylated I53-50 nanoparticles with either 240 glycans (left) or 120 glycans (right) displayed on the particle. The left particle is assembled with 20 glycosylated I53-50A trimeric subunits (protein in gray and glycans in green) and 12 non-glycosylated I53-50B pentameric subunits (orange) to display 240

(legend continued on next page)

week 3 post injection and showing a hierarchy of antibody levels mirroring the glycan composition; antibody responses were strongest against the mannosylated particles with the highest glycan density, with the antibody response against the non-glycosylated subunit (I53-50B) showing a clear trend of increased IgG response with increasing glycan density (Figure S6D). Trends in the antibody response were less clear for the glycosylated trimeric I53-50A subunits, which may reflect the competing effects of glycans promoting FDC localization but also obscuring protein epitopes (Figure S6C). Peak responses were substantially increased for particles with high-mannose glycans compared with particles bearing native complex glycans (Figures S6E and S6F). Altogether, these data suggest that engineered introduction of high-mannose glycans into NP immunogens can be used to direct NPs to follicles and increase the resulting GC response, but that the accessibility of target epitopes must be considered as glycan density increases.

DISCUSSION

Here, we show that MBL- and complement-mediated recognition of glycosylated protein NPs promotes antigen accumulation within draining lymph node follicles, and this accumulation is associated with enhanced humoral responses against a variety of clinically important antigens. The finding that MBL recognition triggers antigen deposition on the FDC network and increased GC responses for multiple, unrelated immunogens and NP scaffolds suggests that this trafficking pathway could provide a generalizable route to enhancing the immunogenicity of vaccines.

By minimally processing and flash-freezing lymph nodes from immunized mice, we were able to clearly visualize eOD and eOD-60mer within the sinuses of lymph nodes early after immunization. Importantly, these data highlight that, at early timepoints, significant amounts of antigen are present within the lymph node regardless of antigen formulation or the presence of MBL. However, 24 hours post immunization, most antigen in the lymph node was localized in the subcapsular and medullary sinuses (Figures 1B and S2A), and the lack of antigen accumulation deeper within the node at later time points when complement recognition is not robust suggests complement receptors may be critical for antigen transfer into the lymph node parenchyma, as has been seen previously in the case of immune complexes (Phan et al., 2007). Interestingly, eOD-60mer in MBL KO mice appears to exhibit a greater degree of medullary localization 1 day post immunization, possibly indicating the presence of an alternative antigen uptake and processing system that is obscured when MBL-mediated trafficking is present (Figures 1B and S2A).

We tested the role of glycan composition on MBL binding and follicular trafficking for two different immunogens, the eOD-60mer and model I53-50 particles. Different forms of MBL are known to have slightly different glycan recognition characteristics, but in general have been described to bind to glycans rich in mannose, glucose, L-fucose, and N-acetylglucosamine subunits (Hansen et al., 2000; Teillet et al., 2005; Zhang et al., 2017). In our studies, we observed that MBL recognition of each NP was dependent on the presence of high-mannose glycans on the particle surface. While our studies do not rule out the possibility of enabling MBL-mediated trafficking through glycosylation with other MBL-recognizable glycans, they highlight the specificity with which MBL recognizes mannosylated surfaces, particularly as compared with surfaces bearing glycans not tailored for MBL binding. However, eOD-60mer coated in primarily complex glycans and subsequently treated with endoglycosidase H still exhibited a degree of follicular localization despite this same eOD-60mer treatment having no MBL recognition *in vitro* (Figures 3B–3D). This could be explained by differences in glycan recognition between recombinant versus native MBL, incomplete removal of high-mannose residues by Endo H treatment, or by the engagement of other lectins or glycan receptors *in vivo* such as sialoadhesin and intelectins (Schnaar, 2015; Wesener et al., 2015). Given that both PNGase F and Endo H selectively act on N-linked glycans (Maley et al., 1989) and the near complete ablation of follicular accumulation following PNGase F treatment, it is unlikely that O-linked glycans are a major contributor to trafficking of the examined eOD-60mer glycoforms. Similarly, the lack of impact on MBL binding following neuraminidase treatment to strip sialic acids from eOD-60mer suggests sialic acids are not mediators of MBL binding (Figure S2E). While the presence of other glycan-specific proteins may have some impact on trafficking, the large reduction in follicular accumulation when only complex glycans were present on eOD-60mer demonstrates the critical nature of high-mannose glycans for reliable antigen deposition in follicles.

We observed a role for MBL and/or complement in the humoral response to five different particulate immunogens in this study. FDC localization was present for all five glycosylated particles in

glycans on the particle. The right particle is assembled with 10 glycosylated and 10 non-glycosylated I53-50A trimeric subunits, and 12 non-glycosylated I53-50B pentameric subunits to display 120 glycans. The two-component nature of I53-50 particles (i.e., each particle is composed of 20 trimers and 12 pentamers) enabled titration of glycan densities on the particle through varying the molar ratio of non-glycosylated to glycosylated I53-50A trimeric subunits; glycosylation of I53-50A trimers was either native or high-mannose for each particle formulation.

(B) Mean glycan distances calculated from the particle structure for NPs with titrated levels of total glycans.

(C) BLI analysis of serially glycosylated I53-50 nanoparticles binding to immobilized recombinant murine MBL2 as a function of I53-50 nanoparticle concentration.

(D) The apparent dissociation constant (K_D) of immobilized murine MBL2 binding to each I53-50 high-mannose glycoform was determined by BLI analysis using a global 1:1 binding model applied to the three highest I53-50 concentrations.

(E and F) C57Bl/6 mice (n = 5/group) were immunized with 5 μg I53-50 high-mannose glycan variants and saponin adjuvant. Shown are average intensity Z projections through 360 μm of cleared draining lymph nodes harvested on days 3 and 7 (E, blue, CD35; red, I53-50; scale bars denote 500 μm), and quantification of the percent I53-50 signal found within follicles (F). Error bars indicate SEM; points represent average values between paired draining lymph nodes from one animal; *, p <0.05; ****, p <0.0001, ns = not significant by one-way ANOVA followed by Tukey post hoc test.

(G) Absolute counts of germinal center B cells and antigen-specific germinal center B cells from WT and MBL KO mice 12 days after immunization with 5 μg I53-50 and saponin adjuvant. Error bars indicate SEM; *, p <0.05; ns = not significant by one-way ANOVA followed by Tukey post hoc test.

WT mice, and it was dramatically reduced for all five when either MBL (eOD-60mer, HA-8mer, HPV16 L1, I53-50) or complement (HBsAg) recognition was eliminated. The HIV gp120 antigen eOD-60mer and influenza HA-8mer exhibited the most profound defects in humoral responses, with reduction in both serum IgG responses and GC responses in MBL KO mice. HPV16 L1 also exhibited MBL-dependent localization to FDCs, though there was no observable difference in the antibody titers elicited in WT and MBL KO mice (Figure 5D). This discrepancy observed between GC and IgG responses could be the result of numerous causes, including variable proportions of GC B cells differentiating into plasmablasts or plasma cells rather than memory B cells, different levels of B cell receptor class switching, or simply the inability of ELISA binding assays to distinguish differences in the polyclonality and affinity of the serum antibody response. HBsAg particles behaved differently than the other particles evaluated here, exhibiting no recognition by MBL *in vitro* but still trafficking to lymph node follicles in a complement-dependent manner. Given the retention of follicular trafficking of HBsAg particles in both MBL and μIgM KO animals but the loss of trafficking in C3 KO animals, it appears that these particles may activate complement via the alternative pathway, as has been observed previously in the case of numerous antigens and pathogens (Cortes et al., 2011; Gupta and Tripathy, 2020; Hall et al., 1993; Kimura et al., 2008; Sissons et al., 1980), or by non-MBL lectins such as ficolins (Ichikawa et al., 1996; Matsushita et al., 2002). These data suggest that any innate recognition pathways leading to complement activation by NP immunogens may impact particle trafficking and downstream immune responses, though, given the relative ease with which high-mannose glycans can be added to NP surfaces, MBL-mediated complement activation may be the most widely applicable pathway for engineering purposes.

The effects of MBL deficiency on humoral immunity observed here likely reflect multiple factors. In addition to altering antigen trafficking, MBL activation of complement may impact B cell activation through engagement of complement receptors, which can provide important costimulatory cues to antigen-specific B cells (Alper et al., 2003; Hellerud et al., 2010; O'Neil et al., 1988). In addition, MBL and complement binding to immunogens could in principle alter epitope accessibility to cognate B cells (Ringe et al., 2019). The relative importance of each of these factors may be antigen-dependent and will be an interesting area for future work. Complement deficiencies have been implicated in reduced antibody production in multiple settings, including both baseline production levels in unimmunized animals and in response to immunization (Alper et al., 2003; Carroll, 2004; Hellerud et al., 2010; O'Neil et al., 1988; Salehen and Stover, 2008). It is therefore likely that complement knockout models exhibit reduced immune responses for multifactorial reasons, with the lack of trafficking observed in the present study being one of these. Alternative factors, such as reduced opsonization, altered cytokine production, and reduced phagocytosis are all possible contributors to the differences in overall measured GC and antibody responses reported here. Despite this, the changes in immune outcomes observed following changes to only the glycan profile of NPs clearly indicate that the presence or absence of follicular accumulation is a key factor in determining the magnitude and nature of the humoral response to these antigen formulations.

Our studies of a systematic series of glycosylated self-assembling I53-50 NPs provide insight into the minimum mannose density required for robust follicular accumulation. Titrating the mean glycosylation of these particles from zero to 240 high-mannose glycans per NP resulted in steady increases in *in vitro* MBL binding as well as *in vivo* FDC localization. These experiments provide an estimate of the minimal glycan patch density required to initiate MBL binding, which for the model ~25 nm particles studied here was 2.1×10^{-3} mannose patches/nm^2, corresponding to a mean separation of ~21 nm between patches. In this model system, the degree of follicular accumulation again correlated with immunogenicity, with FDC-localizing NPs bearing 120 glycans generating significantly greater GC responses than non-trafficking NPs bearing no glycans and mannosylated NPs eliciting stronger antibody responses than natively glycosylated NPs bearing the same number of total glycans. Interestingly, there was a significant drop in antigen density in the FDC networks between day 3 and day 7 for particles bearing less than the maximal 240 glycans per particle. It remains to be determined whether this reflects reduced complement binding and/or FDC retention, more rapid antigen clearance by cognate B cells, or differences in particle stability. These data provide guidance for the creation of future mannosylated NP antigens with enhanced follicular targeting and GC responses.

In the present study, we focused on the role of glycosylation in the trafficking of vaccine NPs, but this is only one factor among a number of physical parameters that will impact antigen trafficking and the subsequent immune response. Particle shape, size, surface charge, and surface chemistry (e.g., presence of reactive moieties, or substantial hydrophilic or hydrophobic patches) also likely play an important role in the fate and function of vaccine particles. Our studies focused on a series of spherical protein NPs within a small range of particle diameters (20–50 nm), but recent studies have shown that larger particles between 50 and 100 nm in diameter are retained within FDC networks and promote more robust antigen-specific responses than do particles with diameters smaller than 15 nm (Zhang et al., 2019). While we have previously shown that non-protein NPs can localize to follicles with the addition of surface mannose (Tokatlian et al., 2018), there remain important questions to be answered regarding the optimal geometry and surface composition of NPs to best facilitate FDC localization and retention.

Collectively, these data demonstrate that MBL- and complement-mediated trafficking of NP antigens is dependent on the presence of a sufficiently dense coating of surface mannose and can occur in a variety of different NP compositions. Further, in the majority of these cases, follicular trafficking is correlated with enhanced GC and antibody responses, reinforcing the importance of glycosylation as a key criterion for the design of efficacious NP antigens.

Limitations of the study

The *in vivo* experiments of this study were conducted primarily in well-characterized, inbred murine models. These models likely fail to fully recapitulate the physiological heterogeneity that would be present in a human population and therefore may not

accurately reflect events that occur following administration of glycosylated NPs in humans. The study also used antibody titer and GC B cell population size as the primary immune response readouts, but it did not investigate other potentially relevant metrics such as the degree of B cell receptor somatic hypermutation or T cell responses that may have provided additional insight.

STAR★METHODS

Detailed methods are provided in the online version of this paper and include the following:

- KEY RESOURCES TABLE
- RESOURCE AVAILABILITY
  - Lead contact
  - Materials availability
  - Data and code availability
- EXPERIMENTAL MODELS AND SUBJECT DETAILS
  - Mice
  - Cell lines
- METHOD DETAILS
  - Plasmids
  - eOD immunogen synthesis
  - Influenza, HPV, and HBV immunogens
  - Synthesis of saponin adjuvant
  - Immunizations
  - Whole lymph node imaging
  - Lymph node processing and imaging for immunofluorescence microscopy
  - Germinal center analysis
  - Antibody titer analysis
  - C3 deposition assay
  - Antigen deglycosylation
  - Bio-layer interferometry
  - Lymph node processing and imaging for whole-tissue confocal microscopy
  - Expression and purification of I53-50A and I53-50B proteins
  - *In vitro* I53-50 nanoparticle assembly and purification
  - Dynamic light scattering of I53-50 particles
  - Negative stain electron microscopy
  - I53-50 endotoxin measurements
  - Glycan profiling
- QUANTIFICATION AND STATISTICAL ANALYSIS

SUPPLEMENTAL INFORMATION

Supplemental information can be found online at https://doi.org/10.1016/j.celrep.2021.110217.

ACKNOWLEDGMENTS

This work was supported in part by the NIAID under awards UM1AI100663 (to D.J.II), AI104715 (to D.J.I.), and AI048240 (to D.J.I.); the Koch Institute support (core) grant P30-CA14051 from the National Cancer Institute; the Ragon Institute of MGH, MIT, and Harvard; and the Bill & Melinda Gates Foundation (OPP1156262 to N.P.K and OPP1126258 to K.K.L.). The content is solely the responsibility of the authors and does not necessarily represent the official views of the National Institutes of Health. D.J.I. is an investigator of the Howard Hughes Medical Institute. We thank the Koch Institute Swanson Biotechnology Center for technical support, specifically the flow cytometry, microscopy, and nanotechnology materials core facilities. We also thank the Biophysical Instrumentation Facility at MIT for use of the Octet biolayer interferometry system. We are grateful to Rashmi Ravichandran for production of the I53-50B.4PT1 pentamer, and we thank William Schief (the Scripps Research Institute) for providing the eOD and eOD-60mer sequences. The graphical abstract and Figure S1 were created with BioRender.com.

AUTHOR CONTRIBUTIONS

B.J.R., L.W., J.C.K., I.S., A.A., S.W., and C.C. designed, performed, and analyzed experiments. A.A. prepared and processed flash-frozen lymph nodes for imaging. J.B. synthesized the HA-8mer immunogens. J.C.K. and I.S. synthesized the I53-50 immunogens. B.J.R., J.C.K., N.P.K., and D.J.I. wrote the manuscript.

DECLARATION OF INTERESTS

The eOD immunogens in this paper are included in patent filings from IAVI, the Scripps Research Institute, and MIT by inventors including D.J.I. N.P.K. is a co-founder, shareholder, and chair of the scientific advisory board of Icosavax, Inc. The King laboratory has received an unrelated sponsored research agreement from Pfizer.

Received: July 22, 2021
Revised: November 3, 2021
Accepted: December 14, 2021
Published: January 11, 2022

REFERENCES

Adolf-Bryfogle, J., Labonte, J.W., Kraft, J.C., Shapovalov, M., Raemisch, S., Lütteke, T., DiMaio, F., Bahl, C.D., Pallesen, J., King, N.P., et al. (2021). Growing Glycans in Rosetta: Accurate de novo glycan modeling, density fitting, and rational sequon design. Biorxiv, 2021.09.27.462000. https://doi.org/10.1101/2021.09.27.462000.

Alper, C.A., Xu, J., Cosmopoulos, K., Dolinski, B., Stein, R., Uko, G., Larsen, C.E., Dubey, D.P., Densen, P., Truedsson, L., et al. (2003). Immunoglobulin deficiencies and susceptibility to infection among homozygotes and heterozygotes for C2 deficiency. J. Clin. Immunol. *23*, 297–305. https://doi.org/10.1023/a:1024540917593.

Arunachalam, P.S., Walls, A.C., Golden, N., Atyeo, C., Fischinger, S., Li, C., Aye, P., Navarro, M.J., Lai, L., Edara, V.V., et al. (2021). Adjuvanting a subunit COVID-19 vaccine to induce protective immunity. Nature *594*, 253–258. https://doi.org/10.1038/s41586-021-03530-2.

Bale, J.B., Gonen, S., Liu, Y., Sheffler, W., Ellis, D., Thomas, C., Cascio, D., Yeates, T.O., Gonen, T., King, N.P., and Baker, D. (2016). Accurate design of megadalton-scale two-component icosahedral protein complexes. Science *353*, 389–394. https://doi.org/10.1126/science.aaf8818.

Bergmann-Leitner, E.S., Duncan, E.H., Leitner, W.W., Neutzner, A., Savranskaya, T., Angov, E., and Tsokos, G.C. (2007). C3d-defined complement receptor-binding peptide p28 conjugated to circumsporozoite protein provides protection against *Plasmodium berghei*. Vaccine *25*, 7732–7736. https://doi.org/10.1016/j.vaccine.2007.08.030.

Boes, M., Esau, C., Fischer, M.B., Schmidt, T., Carroll, M., and Chen, J. (1998). Enhanced B-1 cell development, but impaired IgG antibody responses in mice deficient in secreted IgM. J. Immunol. *160*, 4776–4787.

Brouwer, P.J.M., Antanasijevic, A., Berndsen, Z., Yasmeen, A., Fiala, B., Bijl, T.P.L., Bontjer, I., Bale, J.B., Sheffler, W., Allen, J.D., et al. (2019). Enhancing and shaping the immunogenicity of native-like HIV-1 envelope trimers with a two-component protein nanoparticle. Nat. Commun. *10*, 4272. https://doi.org/10.1038/s41467-019-12080-1.

Brouwer, P.J.M., Brinkkemper, M., Maisonnasse, P., Dereuddre-Bosquet, N., Grobben, M., Claireaux, M., de Gast, M., Marlin, R., Chesnais, V., Diry, S., et al. (2021). Two-component spike nanoparticle vaccine protects macaques from

CellPress
OPEN ACCESS

SARS-CoV-2 infection. Cell *184*, 1188–1200.e19. https://doi.org/10.1016/j.cell.2021.01.035.

Carroll, M.C. (2004). The complement system in regulation of adaptive immunity. Nat. Immunol. *5*, 981–986. https://doi.org/10.1038/ni1113.

Chen, G. (2017). VRC 316: A phase I open-label clinical trial to evaluate dose, safety, tolerability, and immunogenicity of an influenza HA ferritin vaccine, alone or in prime-boost regimens with an influenza DNA vaccine in healthy adults. PLos One *14*, e0222178. NCT03186781. https://clinicaltrials.gov/ct2/show/NCT03186781.

Chen, Y., Wilson, R., O'Dell, S., Guenaga, J., Feng, Y., Tran, K., Chiang, C.-I., Arendt, H.E., DeStefano, J., Mascola, J.R., et al. (2016). An HIV-1 Env–antibody complex focuses antibody responses to conserved neutralizing epitopes. J. Immunol. *197*, 3982–3998. https://doi.org/10.4049/jimmunol.1601134.

Cortes, C., Ferreira, V.P., and Pangburn, M.K. (2011). Native properdin binds to Chlamydia pneumoniae and promotes complement activation. Infect Immun. *79*, 724–731. https://doi.org/10.1128/iai.00980-10.

Dempsey, P.W., Allison, M.E.D., Akkaraju, S., Goodnow, C.C., and Fearon, D.T. (1996). C3d of complement as a molecular adjuvant: Bridging innate and acquired immunity. Science *271*, 348–350. https://doi.org/10.1126/science.271.5247.348.

Diamos, A.G., Larios, D., Brown, L., Kilbourne, J., Kim, H.S., Saxena, D., Palmer, K.E., and Mason, H.S. (2019). Vaccine synergy with virus-like particle and immune complex platforms for delivery of human papillomavirus L2 antigen. Vaccine *37*, 137–144. https://doi.org/10.1016/j.vaccine.2018.11.021.

Diemert, D., and McElrath, J. (2015). A phase I, randomized, double-blind, placebo-controlled dosage escalation trial to evaluate the safety and immunogenicity of eOD-GT8 60mer vaccine, adjuvanted in HIV-uninfected, healthy adult volunteers, NCT03547245. https://clinicaltrials.gov/ct2/show/NCT03547245.

Ehrenstein, M.R., and Notley, C.A. (2010). The importance of natural IgM: Scavenger, protector and regulator. Nat. Rev. Immunol. *10*, 778–786. https://doi.org/10.1038/nri2849.

Fries, L., Cho, I., Krähling, V., Fehling, S.K., Strecker, T., Becker, S., Hooper, J.W., Kwilas, S.A., Agrawal, S., Wen, J., et al. (2019). A randomized, blinded, dose-ranging trial of an Ebola virus glycoprotein (EBOV GP) nanoparticle vaccine with Matrix-M^{TM} adjuvant in healthy adults. J. Infect Dis. *222*, 572–582. https://doi.org/10.1093/infdis/jiz518.

Gach, J.S., Mara, K.J.V., LaBranche, C.C., van Gils, M.J., McCoy, L.E., Klasse, P.J., Montefiori, D.C., Sanders, R.W., Moore, J.P., and Forthal, D.N. (2019). Antibody responses elicited by immunization with BG505 trimer immune complexes. J. Virol. *93*. https://doi.org/10.1128/jvi.01188-19.

Gupta, P., and Tripathy, A.S. (2020). Alternative pathway of complement activation has a beneficial role against Chandipura virus infection. Med. Microbiol. Immun. *209*, 109–124. https://doi.org/10.1007/s00430-019-00648-z.

Hall, R.T., Strugnell, T., Wu, X., Devine, D.V., and Stiver, H.G. (1993). Characterization of kinetics and target proteins for binding of human complement component C3 to the surface-exposed outer membrane of Chlamydia trachomatis serovar L2. Infect Immun. *61*, 1829–1834. https://doi.org/10.1128/iai.61.5.1829-1834.1993.

Hansen, S., Thiel, S., Willis, A., Holmskov, U., and Jensenius, J.C. (2000). Purification and characterization of two mannan-binding lectins from mouse serum. J. Immunol. *164*, 2610–2618. https://doi.org/10.4049/jimmunol.164.5.2610.

Heinen, E., Braun, M., Coulie, P.G., Snick, J.V., Moeremans, M., Cormann, N., Kinet-Denoël, C., and Simar, L.J. (1986). Transfer of immune complexes from lymphocytes to follicular dendritic cells. Eur. J. Immunol. *16*, 167–172. https://doi.org/10.1002/eji.1830160211.

Hellerud, B.C., Aase, A., Herstad, T.K., Næss, L.M., Kristiansen, L.H., Trøseid, A.-M.S., Harboe, M., Lappegård, K.T., Brandtzæg, P., Høiby, E.A., and Mollnes, T.E. (2010). Critical roles of complement and antibodies in host defense mechanisms against Neisseria meningitidis as revealed by human complement genetic deficiencies. Infect Immun. *78*, 802–809. https://doi.org/10.1128/iai.01044-09.

Ichikawa, N., Nakata, M., Mizuochi, T., Matsushita, M., Endo, Y., Taira, S., Sato, Y., and Fujita, T. (1996). A novel human serum lectin with collagen- and fibrinogen-like domains that functions as an opsonin (*). J. Biol. Chem. *271*, 2448–2454. https://doi.org/10.1074/jbc.271.5.2448.

Irvine, D.J., and Read, B.J. (2020). Shaping humoral immunity to vaccines through antigen-displaying nanoparticles. Curr. Opin. Immunol. *65*, 1–6. https://doi.org/10.1016/j.coi.2020.01.007.

Jackson, Y., Chappuis, F., Mezger, N., Kanappa, K., and Loutan, L. (2007). High immunogenicity of delayed third dose of hepatitis B vaccine in travellers. Vaccine *25*, 3482–3484. https://doi.org/10.1016/j.vaccine.2006.12.053.

Jardine, J.G., Kulp, D.W., Havenar-Daughton, C., Sarkar, A., Briney, B., Sok, D., Sesterhenn, F., Ereño-Orbea, J., Kalyuzhniy, O., Deresa, I., et al. (2016). HIV-1 broadly neutralizing antibody precursor B cells revealed by germline-targeting immunogen. Science *351*, 1458–1463. https://doi.org/10.1126/science.aad9195.

Jardine, J.G., Ota, T., Sok, D., Pauthner, M., Kulp, D.W., Kalyuzhniy, O., Skog, P.D., Thinnes, T.C., Bhullar, D., Briney, B., et al. (2015). Priming a broadly neutralizing antibody response to HIV-1 using a germline-targeting immunogen. Science *349*, 156–161. https://doi.org/10.1126/science.aac5894.

Jardine, J., Julien, J.-P., Menis, S., Ota, T., Kalyuzhniy, O., McGuire, A., Sok, D., Huang, P.-S., MacPherson, S., Jones, M., et al. (2013). Rational HIV immunogen design to target specific germline B cell receptors. Science *340*, 711–716. https://doi.org/10.1126/science.1234150.

Kanekiyo, M., Wei, C.-J., Yassine, H.M., McTamney, P.M., Boyington, J.C., Whittle, J.R.R., Rao, S.S., Kong, W.-P., Wang, L., and Nabel, G.J. (2013). Self-assembling influenza nanoparticle vaccines elicit broadly neutralizing H1N1 antibodies. Nature *499*, 102–106. https://doi.org/10.1038/nature12202.

Katagiri, W., Lee, J.H., Tétrault, M., Kang, H., Jeong, S., Evans, C.L., Yokomizo, S., Santos, S., Jones, C., Hu, S., et al. (2019). Real-time imaging of vaccine biodistribution using zwitterionic NIR nanoparticles. Adv. Healthc. Mater. *8*, 1900035. https://doi.org/10.1002/adhm.201900035.

Keech, C., Albert, G., Cho, I., Robertson, A., Reed, P., Neal, S., Plested, J.S., Zhu, M., Cloney-Clark, S., Zhou, H., et al. (2020). Phase 1–2 trial of a SARS-CoV-2 recombinant spike protein nanoparticle vaccine. New Engl. J. Med. *383*, 2320–2332. https://doi.org/10.1056/nejmoa2026920.

Kimura, Y., Miwa, T., Zhou, L., and Song, W.-C. (2008). Activator-specific requirement of properdin in the initiation and amplification of the alternative pathway complement. Blood *111*, 732–740. https://doi.org/10.1182/blood-2007-05-089821.

Krenkova, J., Szekrenyes, A., Keresztessy, Z., Foret, F., and Guttman, A. (2013). Oriented immobilization of peptide-N-glycosidase F on a monolithic support for glycosylation analysis. J. Chromatogr. A. *1322*, 54–61. https://doi.org/10.1016/j.chroma.2013.10.087.

Langley, J., Pastural, E., Halperin, S., McNeil, S., ElSherif, M., MacKinnon-Cameron, D., Ye, L., Grange, C., Thibodeau, V., Cailhier, J.-F., et al. (2020). A randomized controlled study to evaluate the safety and reactogenicity of a novel rVLP-based plant virus nanoparticle adjuvant combined with seasonal trivalent influenza vaccine following single immunization in healthy adults 18–50 Years of age. Vaccines *8*, 393. https://doi.org/10.3390/vaccines8030393.

Link, A.J., Wilkins, M.R., Gasteiger, E., Bairoch, A., Sanchez, J.-C., Williams, K.L., Appel, R.D., and Hochstrasser, D.F. (1998). 2-D proteome analysis protocols. Methods Mol. Biol. Clifton N J *112*, 531–552. https://doi.org/10.1385/1-59259-584-7:531.

Link, A., Zabel, F., Schnetzler, Y., Titz, A., Brombacher, F., and Bachmann, M.F. (2012). Innate immunity mediates follicular transport of particulate but not soluble protein antigen. J. Immunol. *188*, 3724–3733. https://doi.org/10.4049/jimmunol.1103312.

Lövgren-Bengtsson, K., and Morein, B. (2000). Methods in Molecular Medicine, Vaccine Adjuvants: Preparation Methods and Research Protocols (Humana Press).

MacLean, B., Tomazela, D.M., Shulman, N., Chambers, M., Finney, G.L., Frewen, B., Kern, R., Tabb, D.L., Liebler, D.C., and MacCoss, M.J. (2010). Skyline: An open source document editor for creating and analyzing targeted

proteomics experiments. Bioinformatics *26*, 966–968. https://doi.org/10.1093/bioinformatics/btq054.

Madhi, S.A., Polack, F.P., Piedra, P.A., Munoz, F.M., Trenholme, A.A., Simões, E.A.F., Swamy, G.K., Agrawal, S., Ahmed, K., August, A., et al. (2021). Respiratory syncytial virus vaccination during pregnancy and effects in infants. Obstet. Gynecol. Surv. *76*, 10–13. https://doi.org/10.1097/01.ogx.0000725680.06683.d3.

Maley, F., Trimble, R.B., Tarentino, A.L., and Plummer, T.H. (1989). Characterization of glycoproteins and their associated oligosaccharides through the use of endoglycosidases. Anal Biochem. *180*, 195–204. https://doi.org/10.1016/0003-2697(89)90115-2.

Manolova, V., Flace, A., Bauer, M., Schwarz, K., Saudan, P., and Bachmann, M.F. (2008). Nanoparticles target distinct dendritic cell populations according to their size. Eur. J. Immunol. *38*, 1404–1413. https://doi.org/10.1002/eji.200737984.

Marcandalli, J., Fiala, B., Ols, S., Perotti, M., Schueren, W.de van der, Snijder, J., Hodge, E., Benhaim, M., Ravichandran, R., Carter, L., et al. (2019). Induction of potent neutralizing antibody responses by a designed protein nanoparticle vaccine for respiratory syncytial virus. Cell *176*, 1420–1431.e17. https://doi.org/10.1016/j.cell.2019.01.046.

Martin, J.T., Cottrell, C.A., Antanasijevic, A., Carnathan, D.G., Cossette, B.J., Enemuo, C.A., Gebru, E.H., Choe, Y., Viviano, F., Fischinger, S., et al. (2020). Targeting HIV Env immunogens to B cell follicles in nonhuman primates through immune complex or protein nanoparticle formulations. Npj Vaccines *5*, 72. https://doi.org/10.1038/s41541-020-00223-1.

Matsushita, M., Kuraya, M., Hamasaki, N., Tsujimura, M., Shiraki, H., and Fujita, T. (2002). Activation of the lectin complement pathway by H-ficolin (hakata antigen). J. Immunol. *168*, 3502–3506. https://doi.org/10.4049/jimmunol.168.7.3502.

Molina, H., Holers, V.M., Li, B., Fung, Y., Mariathasan, S., Goellner, J., Strauss-Schoenberger, J., Karr, R.W., and Chaplin, D.D. (1996). Markedly impaired humoral immune response in mice deficient in complement receptors 1 and 2. Proc. Natl. Acad. Sci. U S A *93*, 3357–3361. https://doi.org/10.1073/pnas.93.8.3357.

Moon, J.J., Suh, H., Li, A.V., Ockenhouse, C.F., Yadava, A., and Irvine, D.J. (2012). Enhancing humoral responses to a malaria antigen with nanoparticle vaccines that expand Tfh cells and promote germinal center induction. Proc. Natl. Acad. Sci. U S A *109*, 1080–1085. https://doi.org/10.1073/pnas.1112648109.

Movsesyan, N., Mkrtichyan, M., Petrushina, I., Ross, T.M., Cribbs, D.H., Agadjanyan, M.G., and Ghochikyan, A. (2008). DNA epitope vaccine containing complement component C3d enhances anti-amyloid-β antibody production and polarizes the immune response towards a Th2 phenotype. J. Neuroimmunol. *205*, 57–63. https://doi.org/10.1016/j.jneuroim.2008.08.016.

Mueller, S.N., Tian, S., and DeSimone, J.M. (2015). Rapid and persistent delivery of antigen by lymph node targeting PRINT nanoparticle vaccine carrier to promote humoral immunity. Mol. Pharmaceut *12*, 1356–1365. https://doi.org/10.1021/mp500589c.

Naud, P.S., Roteli-Martins, C.M., Carvalho, N.S.D., Teixeira, J.C., Borba, P.C.de, Sanchez, N., Zahaf, T., Catteau, G., Geeraerts, B., and Descamps, D. (2014). Sustained efficacy, immunogenicity, and safety of the HPV-16/18 AS04-adjuvanted vaccine. Hum. Vacc. Immunother. *10*, 2147–2162. https://doi.org/10.4161/hv.29532.

O'Neil, K.M., Ochs, H.D., Heller, S.R., Cork, L.C., Morris, J.M., and Winkelstein, J.A. (1988). Role of C3 in humoral immunity. Defective antibody production in C3-deficient dogs. J. Immunol. *140*, 1939–1945.

Pan, J., and Cui, Z. (2020). Self-assembled nanoparticles: Exciting platforms for vaccination. Biotechnol. J. *15*, 2000087. https://doi.org/10.1002/biot.202000087.

Phan, T.G., Green, J.A., Gray, E.E., Xu, Y., and Cyster, J.G. (2009). Immune complex relay by subcapsular sinus macrophages and noncognate B cells drives antibody affinity maturation. Nat. Immunol. *10*, 786–793. https://doi.org/10.1038/ni.1745.

Phan, T.G., Grigorova, I., Okada, T., and Cyster, J.G. (2007). Subcapsular encounter and complement-dependent transport of immune complexes by lymph node B cells. Nat. Immunol. *8*, 992–1000. https://doi.org/10.1038/ni1494.

Pompa-Mera, E.N., Arroyo-Matus, P., Ocaña-Mondragón, A., González-Bonilla, C.R., and Yépez-Mulia, L. (2014). Protective immunity against enteral stages of Trichinella spiralis elicited in mice by live attenuated Salmonella vaccine that secretes a 30-mer parasite epitope fused to the molecular adjuvant C3d-P28. Res. Vet. Sci. *97*, 533–545. https://doi.org/10.1016/j.rvsc.2014.09.010.

Reddy, S.T., Rehor, A., Schmoekel, H.G., Hubbell, J.A., and Swartz, M.A. (2006). In vivo targeting of dendritic cells in lymph nodes with poly(propylene sulfide) nanoparticles. J. Control Release *112*, 26–34. https://doi.org/10.1016/j.jconrel.2006.01.006.

Ringe, R.P., Portillo, V.M.C., Dosenovic, P., Ketas, T.J., Ozorowski, G., Nogal, B., Perez, L., LaBranche, C.C., Lim, J., Francomano, E., et al. (2019). Neutralizing antibody induction by HIV-1 envelope glycoprotein SOSIP trimers on iron oxide nanoparticles may be impaired by mannose binding lectin. J. Virol. *94*, e01883. https://doi.org/10.1128/jvi.01883-19.

Salehen, N., and Stover, C. (2008). The role of complement in the success of vaccination with conjugated vs. unconjugated polysaccharide antigen. Vaccine *26*, 451–459. https://doi.org/10.1016/j.vaccine.2007.11.049.

Schnaar, R.L. (2015). Glycans and glycan-binding proteins in immune regulation: a concise introduction to glycobiology for the allergist. J. Allergy Clin. Immun. *135*, 609–615. https://doi.org/10.1016/j.jaci.2014.10.057.

Shi, L., Takahashi, K., Dundee, J., Shahroor-Karni, S., Thiel, S., Jensenius, J.C., Gad, F., Hamblin, M.R., Sastry, K.N., and Ezekowitz, R.A.B. (2004). Mannose-binding lectin-deficient mice are susceptible to infection with *Staphylococcus aureus*. J. Exp. Med. *199*, 1379–1390. https://doi.org/10.1084/jem.20032207.

Shukla, S., Myers, J.T., Woods, S.E., Gong, X., Czapar, A.E., Commandeur, U., Huang, A.Y., Levine, A.D., and Steinmetz, N.F. (2017). Plant viral nanoparticles-based HER2 vaccine: immune response influenced by differential transport, localization and cellular interactions of particulate carriers. Biomaterials *121*, 15–27. https://doi.org/10.1016/j.biomaterials.2016.12.030.

Singh, A. (2021). Eliciting B cell immunity against infectious diseases using nanovaccines. Nat. Nanotechnol. *16*, 16–24. https://doi.org/10.1038/s41565-020-00790-3.

Sissons, J.G., Oldstone, M.B., and Schreiber, R.D. (1980). Antibody-independent activation of the alternative complement pathway by measles virus-infected cells. Proc. Natl. Acad. Sci. U S A *77*, 559–562. https://doi.org/10.1073/pnas.77.1.559.

Stephens, L.M., and Varga, S.M. (2020). Nanoparticle vaccines against respiratory syncytial virus. Future Virol. *15*, 763–778. https://doi.org/10.2217/fvl-2020-0174.

Takahashi, K., Ip, W.E., Michelow, I.C., and Ezekowitz, R.A.B. (2006). The mannose-binding lectin: A prototypic pattern recognition molecule. Curr. Opin. Immunol. *18*, 16–23. https://doi.org/10.1016/j.coi.2005.11.014.

Teillet, F., Dublet, B., Andrieu, J.-P., Gaboriaud, C., Arlaud, G.J., and Thielens, N.M. (2005). The two major oligomeric forms of human mannan-binding lectin: Chemical characterization, carbohydrate-binding properties, and interaction with MBL-associated serine proteases. J. Immunol. *174*, 2870–2877. https://doi.org/10.4049/jimmunol.174.5.2870.

Tokatlian, T., Read, B.J., Jones, C.A., Kulp, D.W., Menis, S., Chang, J.Y.H., Steichen, J.M., Kumari, S., Allen, J.D., Dane, E.L., et al. (2018). Innate immune recognition of glycans targets HIV nanoparticle immunogens to germinal centers. Science *363*, eaat9120. https://doi.org/10.1126/science.aat9120.

Turner, M.W. (2003). The role of mannose-binding lectin in health and disease. Mol. Immunol. *40*, 423–429. https://doi.org/10.1016/s0161-5890(03)00155-x.

Walls, A.C., Fiala, B., Schäfer, A., Wrenn, S., Pham, M.N., Murphy, M., Tse, L.V., Shehata, L., O'Connor, M.A., Chen, C., et al. (2020). Elicitation of potent neutralizing antibody responses by designed protein nanoparticle vaccines for

SARS-CoV-2. Cell *183*, 1367–1382.e17. https://doi.org/10.1016/j.cell.2020.10.043.

Wang, N., Qiu, C., Chen, M., Liu, T., and Wang, T. (2019). Covering aluminum oxide nanoparticles with biocompatible materials to efficiently deliver subunit vaccines. Vaccines *7*, 52. https://doi.org/10.3390/vaccines7020052.

Weaver, G.C., Villar, R.F., Kanekiyo, M., Nabel, G.J., Mascola, J.R., and Lingwood, D. (2016). In vitro reconstitution of B cell receptor–antigen interactions to evaluate potential vaccine candidates. Nat. Protoc. *11*, 193–213. https://doi.org/10.1038/nprot.2016.009.

Wesener, D.A., Wangkanont, K., McBride, R., Song, X., Kraft, M.B., Hodges, H.L., Zarling, L.C., Splain, R.A., Smith, D.F., Cummings, R.D., et al. (2015). Recognition of microbial glycans by human intelectin-1. Nat. Struct. Mol. Biol. *22*, 603–610. https://doi.org/10.1038/nsmb.3053.

Wessels, M.R., Butko, P., Ma, M., Warren, H.B., Lage, A.L., and Carroll, M.C. (1995). Studies of group B streptococcal infection in mice deficient in complement component C3 or C4 demonstrate an essential role for complement in both innate and acquired immunity. Proc. Natl. Acad. Sci. *92*, 11490–11494. https://doi.org/10.1073/pnas.92.25.11490.

Wibowo, D., Jorritsma, S.H.T., Gonzaga, Z.J., Evert, B., Chen, S., and Rehm, B.H.A. (2020). Polymeric nanoparticle vaccines to combat emerging and pandemic threats. Biomaterials *268*, 120597. https://doi.org/10.1016/j.biomaterials.2020.120597.

Widge, A. (2019). VRC 321: A phase I open-label clinical trial to evaluate dose, safety, tolerability, and immunogenicity of an influenza H1 stabilized stem ferritin vaccine, VRCFLUNPF099-00-VP, in healthy adults.NCT03814720. https://clinicaltrials.gov/ct2/show/NCT03814720.

Zhang, W., Eijk, M.van, Guo, H., Dijk, A.van, Bleijerveld, O.B., Verheije, M.H., Wang, G., Haagsman, H.P., and Veldhuizen, E.J.A. (2017). Expression and characterization of recombinant chicken mannose binding lectin. Immunobiology *222*, 518–528. https://doi.org/10.1016/j.imbio.2016.10.019.

Zhang, Y.-N., Lazarovits, J., Poon, W., Ouyang, B., Nguyen, L.N.M., Kingston, B.R., and Chan, W.C.W. (2019). Nanoparticle size influences antigen retention and presentation in lymph node follicles for humoral immunity. Nano Lett. *19*, 7226–7235. https://doi.org/10.1021/acs.nanolett.9b02834.

STAR★METHODS

KEY RESOURCES TABLE

REAGENT or RESOURCE	SOURCE	IDENTIFIER
Antibodies		
αCD35, clone 8C12, in BV421	BD Biosciences	Cat#740029; RRID: AB2739801
αCD16/CD32, clone 93	ThermoFisher	Cat#14-0161-85; RRID: AB_467133
αCD45R/B220, clone RA3-6B2, in PE/Cy7	BioLegend	Cat#103222; RRID: AB_313005
αCD4, clone RM4-5, in BV711	BioLegend	Cat#100550; RRID: AB_2562099
αGL7, clone GL7, in PerCP/Cy5.5	BioLegend	Cat#144610; RRID: AB_2562979
αCD38, clone 90, in AF488	BioLegend	Cat#102714; RRID: AB_528796
αC3, clone 11H9	Abcam	Cat#ab11862; RRID: AB_1119819
α-sialic acid, polyclonal	LSBio	Cat#LS-C664155
Chemicals, peptides, and recombinant proteins		
Recombinant mouse MBL2	R&D Systems	Cat#2208-MB-050/CF
Recombinant HPV16 L1	Abcam	Cat#ab119880
Recombinant HBsAg AD	Abcam	Cat#ab193473
Cholesterol (ovine)	Avanti Polar Lipids	Cat#700000
16:0 PC (DPPC)	Avanti Polar Lipids	Cat#850355
N-Decanoyl-N-methylglucamine (MEGA-10)	Sigma-Aldrich	Cat#D6277
Quil-A	Invivogen	Cat#vac-quil
LIVE/DEAD fixable aqua dead cell stain kit	ThermoFisher	Cat#L34957
PNGase F	New England Biolabs	Cat#P0704S
Endoglycosidase H	New England Biolabs	Cat#P0702S
α2-3,6,8,9 Neuraminidase A	New England Biolabs	Cat#P0722S
Erythrina cristagalli gel immobilized lectin	EY Laboratories	Cat#A-5901-2
D-lactose monohydrate	Sigma-Aldrich	Cat#61345
Critical commercial assays		
Limulus amebocyte lysate assay	Lonza	Cat#QCL-1000
Cholesterol quantification kit	Sigma-Aldrich	Cat#MAK043
Alexa Fluor 647 antibody labeling kit	ThermoFisher	Cat#A20186
Experimental models: Cell lines		
Expi293F cells	ThermoFisher	Cat#A14527
FreeStyle 293-F cells	ThermoFisher	Cat#R79007
Experimental models: Organisms/strains		
Mouse: wild-type: C57Bl/6J	The Jackson Laboratory	JAX: 000664
Mouse: MBL KO: B6.129S4-$Mbl1^{tm1Kata}Mbl2^{tm1Kata}$/J	The Jackson Laboratory	JAX: 006122
Mouse: C3 KO: B6;129S4-$C3^{tm1Crr}$/J	The Jackson Laboratory	JAX: 003641
Mouse: Cr1/2 KO: B6.129S7(NOD)-$Cr2^{tm1Hmo}$/J	The Jackson Laboratory	JAX: 008225
Mouse: wild-type: B6129SF2/J	The Jackson Laboratory	JAX: 101045
Mouse: μIgM KO: B6;129S4-$Ighm^{tm1Che}$/J	The Jackson Laboratory	JAX: 003751
Recombinant DNA		
pHLSec-eOD	Jardine et al. (2013)	N/A
pHLSec-eOD-60mer	Jardine et al. (2013)	N/A
pCMV/R-mcs-HA-8mer	Kanekiyo et al. (2013)	N/A
pET29b-I53-50A	Bale et al. (2016)	N/A
pET29b-I53-50B.4PT1	Bale et al. (2016)	N/A
pCMV/R-I53-50A_4gly	This paper	N/A

(*Continued on next page*)

Continued

REAGENT or RESOURCE	SOURCE	IDENTIFIER
Software and algorithms		
Prism version 9.2.0	GraphPad	https://www.graphpad.com/scientific-software/prism/
Octet Data analysis software version 8.1	ForteBio	https://www.sartorius.com/en/products/protein-analysis/octet-systems-software
FACSDiva	BD Biosciences	https://www.bdbiosciences.com/en-eu/products/software/instrument-software/bd-facsdiva-software#Overview
FlowJo version 10	BD Biosciences	https://www.flowjo.com/solutions/flowjo
ImageJ	NIH	https://imagej.nih.gov/ij/

RESOURCE AVAILABILITY

Lead contact

Further information and requests for resources and reagents should be directed to and will be fulfilled by the lead contact Darrell J. Irvine (djirvine@mit.edu).

Materials availability

Material transfer agreements with standard academic terms will be established to document sharing of reagents developed in this study.

Data and code availability

- All data reported in this paper will be shared by the lead contact upon reasonable request.
- This paper does not report original code.
- Any additional information required to reanalyze the data reported in this paper is available from the lead contact upon request.

EXPERIMENTAL MODELS AND SUBJECT DETAILS

Mice

C57BL/6 (stock no. 000664), MBL KO (stock no. 006122) (Shi et al., 2004), C3 KO (stock no. 003641) (Wessels et al., 1995), Cr1/2 KO (stock no. 008225) (Molina et al., 1996), B6129SF2/J (stock no. 101045), and μIgM KO (stock no. 003751) (Boes et al., 1998) mice were purchased from Jackson Laboratory (Bay Harbor, ME) and were housed in animal facilities at the Massachusetts Institute of Technology. Mice were housed under specific pathogen-free conditions. All procedures used in this study were approved by the Committee on Animal Care at the Massachusetts Institute of Technology following local, state, and federal regulations. Adult female mice age 6 to 10 weeks used for all studies, except in limited cases when availability required the use of a small number of male mice.

Cell lines

All proteins not obtained directly from suppliers were expressed in human embryonic kidney cells. Expi293F cells were purchased from ThermoFisher Scientific and were cultured in Expi293 Expression Medium (ThermoFisher Scientific A1435101) to a density of 3.0×10^6 cells per mL at 37°C, 70% humidity, 8% CO_2, and rotating at 150 rpm. FreeStyle 293-F cells were purchased ThermoFisher Scientific and were cultured in Gibco Freestyle 293 Expression Medium (ThermoFisher Scientific 12338018) to a density of 1.2×10^6 cells per mL at 37°C, 78% humidity, 8% CO_2, and rotating at 120 rpm.

METHOD DETAILS

Plasmids

Previously developed plasmids were used to express eOD monomer, eOD-60mer, HA-8mer, I53-50A, and I53-50B (Bale et al., 2016; Jardine et al., 2013; Kanekiyo et al., 2013). The plasmid for the glycosylated I53-50A trimer encoding the four sequon-introducing substitutions NAT, YANET, NFT, and FHNAT is newly generated here and the final construct contained a N-terminal secretion signal sequence derived from the modified bovine prolactin (MDSKGSSQKGSRLLLLLVVSNLLLPQGVLA) and C-terminal myc and hex-histidine tags (LEEQKLISEEDLHHHHHH). This construct was then cloned by GenScript into the pCMV/R plasmid using the restriction sites XbaI and AvrII.

eOD immunogen synthesis

eOD monomer and eOD-60mer were synthesized as previously reported (Jardine et al., 2013, 2015). Briefly, for eOD monomer synthesis, plasmids were transiently transfected into Expi293F cells. After 5 days of culturing in conditions described above, cell culture supernatants were collected and protein was purified in an ÄKTA pure chromatography system using HiTrap HP Ni sepharose affinity column, followed by size exclusion chromatography using Superdex 75 Increase 10/300 GL column (GE Healthcare). Endotoxin levels in purified protein was measured using Endosafe Nexgen-PTS system (Charles River) and were < 5EU/mg protein. eOD-60mer was produced via the same method with the following modifications: (1) unless otherwise indicated, kifunensine was included in the cell media; (2) the affinity chromatography step was done by overnight 4°C incubation on Galanthus Nivalis Lectin agarose beads (Vector Laboratories #AL-1243), elution with Lectin Elution Buffer (1M Methyl a-D-mannopyranoside) followed by dialysis into PBS, and; (3) the size exclusion chromatography was performed used a Superose 6 column (GE Healthcare). Particle formation was assessed by SECMALS and DLS analysis.

Influenza, HPV, and HBV immunogens

HA-8mer was synthesized as previously described (Kanekiyo et al., 2013; Weaver et al., 2016). Briefly, plasmids were transiently transfected into FreeStyle 293-F cells in FreeStyle 293 Expression Medium. After 5 days of culturing in conditions described above, cell culture supernatants collected by centrifugation and concentrated using a tangential flow filtration setup with a 30 kDa cutoff. In 100 mL aliquots, the concentrate was mixed with 2 mL of PBS-equilibrated *Erythrina cristagalli* lectin-immobilized resin (EY Laboratories) at 4°C and incubated overnight with gentle agitation. The resin was then loaded onto a 1.5 × 20 cm glass Econo-Column (Bio-Rad) and washed with five column-volumes of PBS by gravity flow. HA-8mer particles were eluted with two column-volumes of 0.2 M D-lactose (Sigma-Aldrich) in PBS and concentrated in a centrifugal concentrator with a 100 kDa cutoff. Size-exclusion FPLC was then performed using a Superdex 200 10/30 column (GE Healthcare) and purified HA-8mer was again concentrated as before.

Recombinant HPV16 L1 (Abcam ab119880) and recombinant HBsAg AD (Abcam ab193473) were reconstituted following the manufacturer's guidelines. Nanoparticle formation of the expected size was confirmed by dynamic light scattering.

Synthesis of saponin adjuvant

The adjuvant used for all the described studies was an ISCOM-like nanoparticle comprised of self-assembled cholesterol, phospholipid, and Quillaja saponin prepared as previously described (Lövgren-Bengtsson and Morein, 2000); all synthesis was performed under sterile conditions with sterile reagents. Briefly, 10 mg each of cholesterol (Avanti Polar Lipids 700000) and DPPC (Avanti Polar Lipids 850355) were dissolved separately in 20% MEGA-10 (Sigma D6277) detergent at a final concentration of 20 mg/mL and 50 mg Quil-A saponin (Invivogen vac-quil) was dissolved in MQ water at a final concentration of 100 mg/mL. Next, DPPC solution was added to cholesterol followed by addition of Quil-A saponin in rapid succession and the volume was brought up with PBS to a final concentration of 1 mg/mL cholesterol and 2% MEGA-10. The solution was allowed to equilibrate at 25°C overnight, followed by 5 days of dialysis against PBS using a 10k MWCO membrane. The adjuvant solution was then filter sterilized using a 0.2 μm Supor syringe filter, concentrated using 50k MWCO centricon filters, and further purified by FPLC using a Sephacryl S-500 HR size exclusion column. Each adjuvant batch was finally characterized by negative stain TEM and DLS to confirm uniform morphology and size and validated for low endotoxin by Limulus Amebocyte Lysate assay (Lonza QCL-1000). Final adjuvant concentration was determined by cholesterol quantification (Sigma MAK043).

Immunizations

Mice age 6 to 10 weeks of age were immunized with immunogen and adjuvant via subcutaneous tail-base injection with 50 μL on either side of the tail for a total of 100 μL per animal. Mice immunized with eOD or eOD-60mer received a total of 2 μg eOD, mice immunized with HPV16 L1 received 0.1 μg total protein, and mice immunized with HA-8mer, HBsAg, and I53-50 received 5 ug total protein. All immunizations included 5 μg saponin adjuvant. For trafficking studies, mice were similarly immunized with AlexaFluor 647-tagged immunogens labeled following the manufacturer's instructions (ThermoFisher A20186). Immunogens were characterized by UV-vis spectroscopy and contained approximately 1 dye per monomeric eOD, 40 dyes per eOD-60mer particle, 45 dyes per HA-8mer particle, and 50 dyes per HPVL1 16, HBsAg, and I53-50 particle. Mice were then injected subcutaneously in the tail base with 4 μg BV421-labeled anti-CD35 (BD Biosciences 740029) 18 to 24 h prior to lymph node excision to label follicles *in situ*.

Whole lymph node imaging

Mice were euthanized and both inguinal lymph nodes were harvested at the specified days. Lymph nodes were immediately placed into PBS containing 4% paraformaldehyde overnight and were then washed twice in PBS. Imaging was performed using an IVIS spectrum optical imaging system. All lymph nodes were imaged at the same time using automatically determined imaging settings based on the fluorescence of the nodes. Background subtraction was performed based on an empty portion of the image. The MFI of each pair of lymph nodes was determined using an identically sized gate, and the MFI of a pair of lymph nodes from an unimmunized mouse was subtracted from the MFIs of experimental mice to correct for lymph node autofluorescence.

Lymph node processing and imaging for immunofluorescence microscopy

Lymph nodes were excised from mice and immediately placed in OCT. Nodes were then flash frozen using liquid nitrogen to prevent antigen leakage. Frozen lymph node blocks were sliced into 100 μm thick sections using a Leica CM1950 cryostat. For each node, six slices were obtained and mounted onto slides. Slides were stored at −80°C. Lymph nodes were imaged using a Leica SP8 Laser Scanning Confocal Microscope. Lasers were set to minimize pixel saturation in the brightest samples in each experiment. All laser and channel settings were then kept constant across each individual studies to allow for direct comparison between different samples.

Germinal center analysis

Mice were sacrificed by carbon dioxide inhalation and both inguinal lymph nodes were harvested at the specified days. Lymph nodes were processed into single-cell suspensions by mechanical digestion followed by passage through a 70 μm cell strainer (BD Biosciences) twice. Next, cells were washed with PBS and stained with Live/Dead Aqua (ThermoFisher L34957) for 15 min at room temperature. Samples were then treated with anti-CD16/32 Fc block (ThermoFisher14-0161-85), followed by staining with anti-B220-PE-Cy7 (BioLegend 103222), anti-CD4-BV711 (BioLegend 100550), anti-GL7-PerCP/Cy5.5 (BioLegend 144610), anti-CD38-AF488 (BioLegend 102714), and antigen-bearing nanoparticles separately bearing Pacific Blue (ThermoFisher P30013) and AF647. Excess staining reagents were washed off and cells were analyzed via a BD FACSCelesta flow cytometer.

Antibody titer analysis

Blood samples were collected from immunized mice via retro-orbital bleeds and serum was isolated. MaxiSorp plates (ThermoFisher 44-2404-21) were coated with 2 μg/mL immunogen and blocked overnight in PBS containing 1% BSA. Plates were washed four times in PBS containing 0.2% Tween-20, and dilutions of serum in blocking buffer were added and incubated for two hours. Plates were washed as before and an HRP-conjugated anti-mouse IgG was added and incubated for one hour. Plates were washed and TMB was added. The reaction was stopped with sulfuric acid once the wells containing the lowest dilutions of TMB began to develop visually or after 20 min and the absorbance of each well was determined. All titers reported are inverse dilutions where $A_{450nm} - A_{540nm}$ (reference wavelength) equals 0.5, with the exception of data from I53-50 immunizations, which is reported as area under the curve (AUC) measurements to better differentiate between the observed responses.

C3 deposition assay

High-mannose eOD-60mer was coated directly on to MaxiSorp plates in 50 μL PBS at 3 μg/mL eOD. Plates were then blocked overnight in PBS containing 1% BSA and 0.1 M CaCl. 30% fresh wild-type or MBL KO mouse serum in PBS was added and plates were incubated at 37 C for two hours. Plates were then washed four times in PBS containing 0.1% Tween 20 and anti-C3 antibodies were added, followed by another 2 h incubation at room temperature. Plates were washed as before and HRP-conjugated secondary antibodies were added. Following an hour-long incubation and additional washes, TMB was added and plates were developed until the lowest dilution wells started to visually show signal. The reaction was then stopped with sulfuric acid and the absorbance at 450 nm was read via plate reader.

Antigen deglycosylation

Nanoparticle immunogens were deglycosylated using PNGase F (New England BioLabs P0704S), endoglycosidase H (New England BioLabs P0702S), or α2-3,6,8,9 neuraminidase A (New England BioLabs P0722S) under non-denaturing conditions following the manufacturer's guidelines. Deglycosylation was confirmed via SDS-PAGE gel using glycoprotein stain (ThermoFisher 24562). Retention of particle structure was confirmed via dynamic light scattering.

Bio-layer interferometry

All bio-layer interferometry measurements were conducted using a ForteBio Octet RED96 instrument in the MIT Biophysical Instrumentation Facility. Streptavidin-coated sensors were incubated in PBS containing 1% BSA and 0.1 M CaCl and were then loaded into wells of the same solution containing 1 μg/mL biotinylated murine MBL2 (R&D Systems 2208-MB-050/CF) for 1 min. Excess MBL was washed off and MBL-coated biosensors were moved to wells containing dilutions of antigen formulations until probes began to become saturated. The biosensors were then moved back to the baseline solution and antigen was allowed to dissociate.

Lymph node processing and imaging for whole-tissue confocal microscopy

Lymph nodes were excised from mice and fixed overnight at 4°C in 4% paraformaldehyde. Lymph nodes were then processed as previously described (Tokatlian et al., 2018). Briefly, nodes were washed twice in PBS and excess fat and connective tissue were removed. Nodes were then gradually moved into solutions containing successively high concentrations of methanol over the course of several hours until they were incubated for half an hour in pure methanol. Nodes were then briefly bleached in hydrogen peroxide solution for 1 min before being returned to methanol for half an hour. They were then gradually moved into solutions containing increasing concentrations of tertiary-butanol before being incubated in pure teritary-butanol for one hour. All solutions used after bleaching contained an additional 0.4% α-tocopherol. Nodes were then removed from solution and allowed to dry completely before

being placed in dichloromethane. After lymph nodes dropped to the bottom of tubes following swirling, indicating the removal of remaining tertiary-butanol, they were stored in dibenzyl ether, which was used as an optical clearing solution.

Lymph nodes were imaged using an Olympus FV1200 Laser Scanning Confocal Microscope. Lasers were set to minimize pixel saturation in the brightest samples in each experiment. All laser and channel settings were then kept constant across each individual studies to allow for direct comparison between different samples. Each lymph node was imaged over 360 μm. For studies comparing the total number of antigen-positive follicles, channel settings were increased to allow for imaging through the entire height of the lymph node regardless of signal saturation.

Microscopy images were analyzed using ImageJ as previously described (Tokatlian et al., 2018). To reduce background signal bleed, the antigen channel was passed through an HSB filter against background fluorescence. Z-stacks were then condensed into average intensity projections ranging over the full 360 μm displaying the average intensity of each color for each pixel.

To quantify immunogen signal and follicle colocalization, the maximum intensity z-projection on all channels was binarized and used to define a selection comprising the whole lymph node area. Next, for each z slice a high-pass filter was applied such that the brightest pixels in the background autofluorescence channel were binarized. These bright pixels in the autofluorescence channel were zeroed in the signal channels to mitigate the intensity effects of bleed-through from autofluorescence into the signal channels. A sum intensity z-projection on the CD35 channel was binarized and used to define a selection comprising the follicular area. A sum intensity z-projection on the immunogen channel was then binarized using a high pass filter such that bright pixels were applied an intensity value of 1, while dim pixels were applied an intensity value of 0. This binary mask was used to multiply a sum intensity z-projection such that all dim pixels were zeroed while all bright pixels retained their unaltered intensity information. The intensity of these bright pixels was measured within both the whole lymph node and follicular areas for use in ratiometric antigen signal intensity calculations.

Expression and purification of I53-50A and I53-50B proteins

To generate glycosylated I53-50A trimers (Adolf-Bryfogle et al., 2021; Bale et al., 2016), an expression plasmid encoding the sequon-introducing substitutions NAT, YANET, NFT, and FHNAT was synthesized by Genscript. For large-scale glycosylated and non-glycosylated I53-50A protein expression, Expi293F cells transiently transfected using PEI-MAX (Polyscience). For production of I53-50A bearing high-mannose glycans, kifunensine dissolved in PBS was added to the expression medium to reach a final concentration of 10 μM. Supernatants were clarified by centrifugation (5 min at 4000 rcf), PDADMAC solution was added to a final concentration of 0.0375% (Sigma Aldrich, #409014), and a final spin was performed (5 min at 4000 rcf). Proteins were purified from clarified supernatants via a batch bind method where Talon cobalt affinity resin (Takara) was added to supernatants and allowed to incubate for 15 min with gentle shaking. Resin was isolated using 0.2 μm vacuum filtration and transferred to a gravity column, where it was washed with 20 mM Tris pH 8.0, 300 mM NaCl, and protein was eluted with 3 column volumes of 20 mM Tris pH 8.0, 300 mM NaCl, 300 mM imidazole. This batch bind process was repeated a second time on the supernatant flow-through from the filtration step. Eluate with protein was concentrated to ~2 mL using a 30 kDa MWCO Amicon concentrator (Millipore Sigma). The concentrated sample was sterile filtered (0.2 μm) and applied to a Superdex 200 Increase 10/300 SEC column (Cytiva) using 25 mM Tris pH 8.0, 150 mM NaCl, 0.75% CHAPS, 5% glycerol buffer. The I53-50B.4PT1 pentamer was produced and purified as previously described Bale et al., (2016).

In vitro I53-50 nanoparticle assembly and purification

The protein concentration of individual nanoparticle components (I53-50A trimer and I53-50B pentamer) was determined by measuring 280 nm absorbance using a UV/vis spectrophotometer (Agilent Cary 8454) and estimated extinction coefficients(Link et al., 1998). Particle assembly was performed by adding equimolar amounts of I53-50A and I53-50B to reach a final protein concentration of 20 μM (10 μM for each individual component) and resting on ice for at least 30 min. Particles of varying glycan density were prepared by using mixtures of glycosylated and non-glycosylated I53-50A trimers at the desired ratios in these *in vitro* assembly reactions. The target ratio represents the bulk average of glycosylated to non-glycosylated trimeric building blocks in the resultant nanoparticles; the number of glycosylated trimers in each individual nanoparticle will be normally distributed around this target due to random incorporation of each type of trimer during assembly. Assembled particles were sterile filtered (0.2 μm) immediately before SEC purification using a Superose 6 Increase 10/300 GL column to remove residual unassembled component.

Dynamic light scattering of I53-50 particles

Dynamic light scattering (DLS) was used to measure the hydrodynamic diameter and polydispersity of I53-50 nanoparticles on an UNcle (UNchained Laboratories). 8.8 μL of 0.1 mg/mL protein was applied to a quartz capillary cassette (UNi, UNchained Laboratories) to obtain intensity measurements from 10 acquisitions of 5 s each, using auto-attenuation of the laser. Increased viscosity due to 5% glycerol in the buffer was accounted for by the UNcle software.

Negative stain electron microscopy

A sample volume of 3 μL at a concentration of 70 μg/mL protein in 50 mM Tris pH 8, 150 mM NaCl, 5% v/v glycerol was applied to a freshly glow-discharged 300-mesh copper grid (Ted Pella) and incubated on the grid for 1 min. The grid was then dipped in a 40 μL droplet of water, excess liquid was blotted away with filter paper (Whatman), the grid was dipped into 3 μL of 0.75% w/v uranyl

formate stain, stain was immediately blotted off with filter paper, then the grid was dipped again into another 3 μL of stain and incubated for ~30 s. Finally, the stain was blotted away and the grids were allowed to dry for 1 min prior to storage or imaging. Prepared grids were imaged in a Talos model L120C transmission electron microscope using a Gatan camera at 57,000×.

I53-50 endotoxin measurements

Endotoxin levels in I53-50 samples were measured using the EndoSafe Nexgen-MCS System (Charles River). Samples were diluted 1:100 in Endotoxin-free LAL reagent water, and applied into wells of an EndoSafe LAL reagent cartridge. Endotoxin content was analyzed using Charles River EndoScan-V software, which automatically back-calculates for the 1:100 dilution factor. Endotoxin values reported as EU/mL were converted to EU/mg based on protein concentration obtained by UV-Vis measurements. All endotoxin values were <20 EU/mg.

Glycan profiling

A bottom-up mass spectrometry (MS) approach was used to identify I53-50A trimer N-glycosylation profiles. Aliquots of 1mg/mL + Kif and -Kif I53-50A protein were denatured in a 25 mM Tris buffer (pH 8.0) containing 7 M guanidinium chloride (GdnHCl) and 50 mM dithiothreitol (DTT) at 90°C for 30 min. The solution was mixed with 100 mM fresh iodoacetamide (IAA) and incubated at 25°C for 1 h in the dark to alkylate the reduced cysteines. 50 mM DTT was then added to quench the excess IAA. The GndHCl concentration was diluted 11-fold to about 0.6 M by adding 10 mM Tris (pH 8.0), 2 mM calcium chloride solution. Half reaction solution (280 μL) was separated and treated with 10 units recombinant Peptide N-glycanase F (GST-PNGase F) (Krenkova et al., 2013) at 37°C for 1 h to convert glycosylated asparagines to aspartic acids. Both PNGase F treated and untreated samples were then digested with Glu-C (Promega) at a ratio of 1:40 (w/w) overnight at 37°C. The digested samples were quenched by 0.02% formic acid (FA), followed by desalting by Sep-Pak C18 cartridges (Waters). All the water and organic solvents used were MS grade (Optima™, Fisher).

Glycopeptide data collection was performed by nano LC-MS using an Orbitrap Fusion™ mass spectrometer. A 2 cm trapping column and a 35 cm analytical column were freshly prepared in fused silica (100 μm ID) with 5 μM ReproSil-Pur C18 AQ beads (Dr. Maisch). 8 μL sample was run by a 60-min linear gradient from 2% to 30% acetonitrile (ACN) with 0.1% FA, followed by 10-min 80% ACN wash. An EThcD method was optimized with following settings: ion source: 2.1 kV for positive mode; resolution MS^1 = 120000, MS^2 = 30000; AGC target: MS^1 = $2e^5$, MS^2 = $1e^5$; and injection time: MS^1 = 50 ms, MS^2 = 60 ms.

Glycopeptide data were visualized and processed by Byonic™ (Version 3.8, Protein Metrics Inc.) and Skyline(MacLean et al., 2010) (MacCoss Lab, University of Washington) using the 6 ppm precursor and 10 ppm fragment mass tolerance. Glycopeptides were matched using the N-glycan 309 mammalian database in Byonic™ and validated by correct c- and z- fragment ions as well as glycan oxonium ions. The relative abundance of each glycoform was determined from peak area using Skyline software. Glycoforms were identified as either Oligomannose: HexNAc(2)Hex(9–5); Hybrid: HexNAc(3)Hex(5–6); or Complex: HexNAc(3)Hex(3–4)X and HexNAc(4–6)X with or without fucosylation.

QUANTIFICATION AND STATISTICAL ANALYSIS

All biolayer interferometry assays were analyzed using the Octet data analysis software (ForteBio, version 8.1) and were visualized using GraphPad Prism version 9.2. Statistical analyses were performed using GraphPad Prism version 9.2 software. All values and errors bars are shown as mean ± standard error. Data was analyzed using Mann-Whitney tests or ordinary one-way ANOVA followed by a Tukey's post-test to compare multiple groups, with this information, sample group sizes, and statistical significance cutoffs noted in figure captions.

Cell Reports, Volume *38*

Supplemental information

Mannose-binding lectin and complement mediate follicular localization and enhanced immunogenicity of diverse protein nanoparticle immunogens

Benjamin J. Read, Lori Won, John C. Kraft, Isaac Sappington, Aereas Aung, Shengwei Wu, Julia Bals, Chengbo Chen, Kelly K. Lee, Daniel Lingwood, Neil P. King, and Darrell J. Irvine

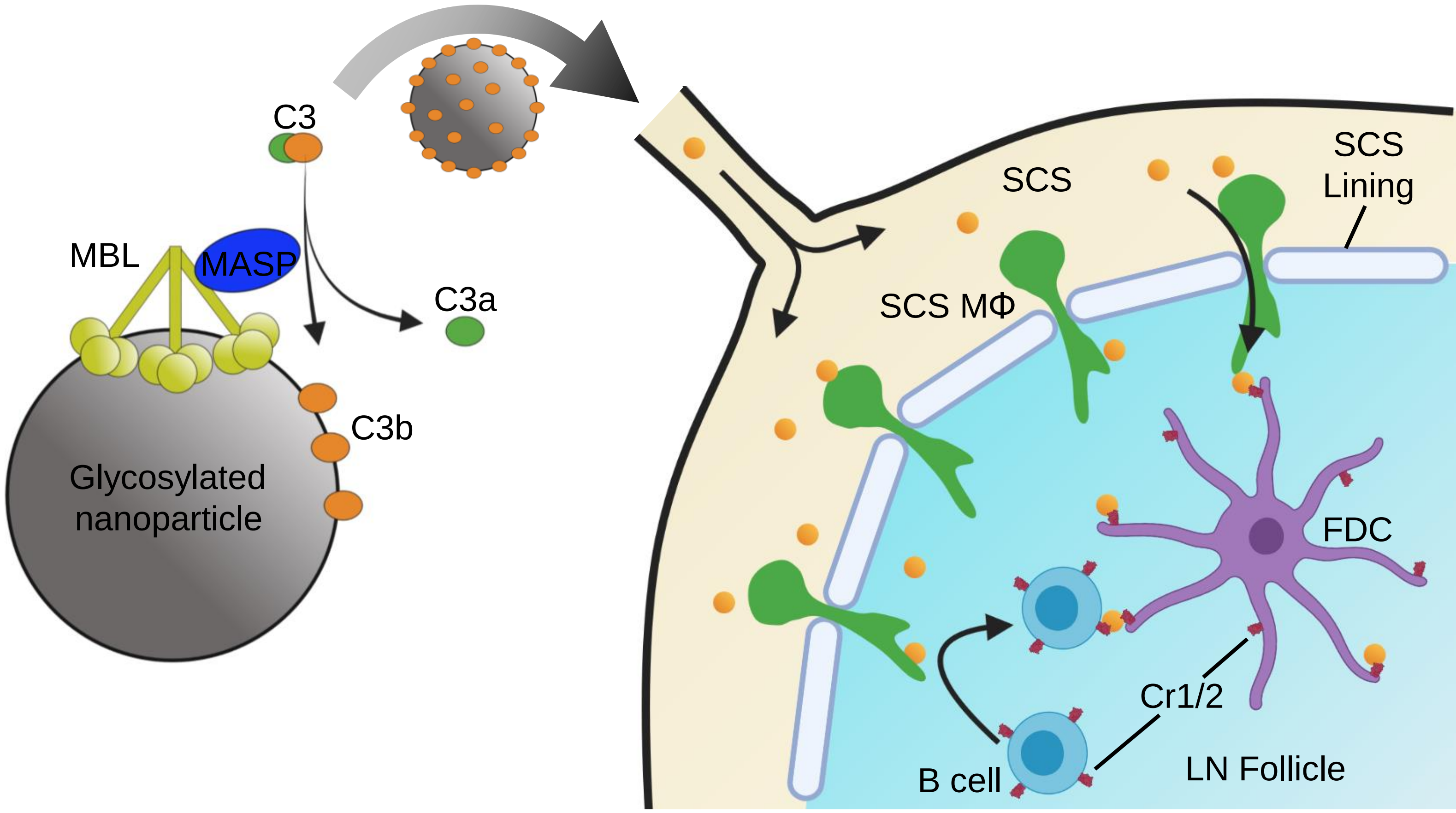

Figure S1. Schematic of MBL-mediated nanoparticle trafficking pathway. Related to Figure 1. Heavily glycosylated nanoparticulate antigens are bound by MBL following injection. Upon MBL binding, MBL-associated serine proteases (MASPs) become active and either directly or indirectly cleave C3 into components C3a and C3b, the latter of which opsonizes the nanoparticle. Opsonized nanoparticles enter the lymph node from afferent lymphatic vessels into the subcapsular sinus (SCS) and are bound by subcapsular sinus macrophages (SCS MΦ), which transport particles to the interior of the node through translocation and/or transcytosis. The nanoparticles are then transported to follicular dendritic cells (FDCs) by B cells, via cognate B cell receptor recognition or noncognate complement receptor (Cr1 and Cr2) binding, or by direct binding of an FDC dendrite, where they are captured and displayed by Cr1 and Cr2.

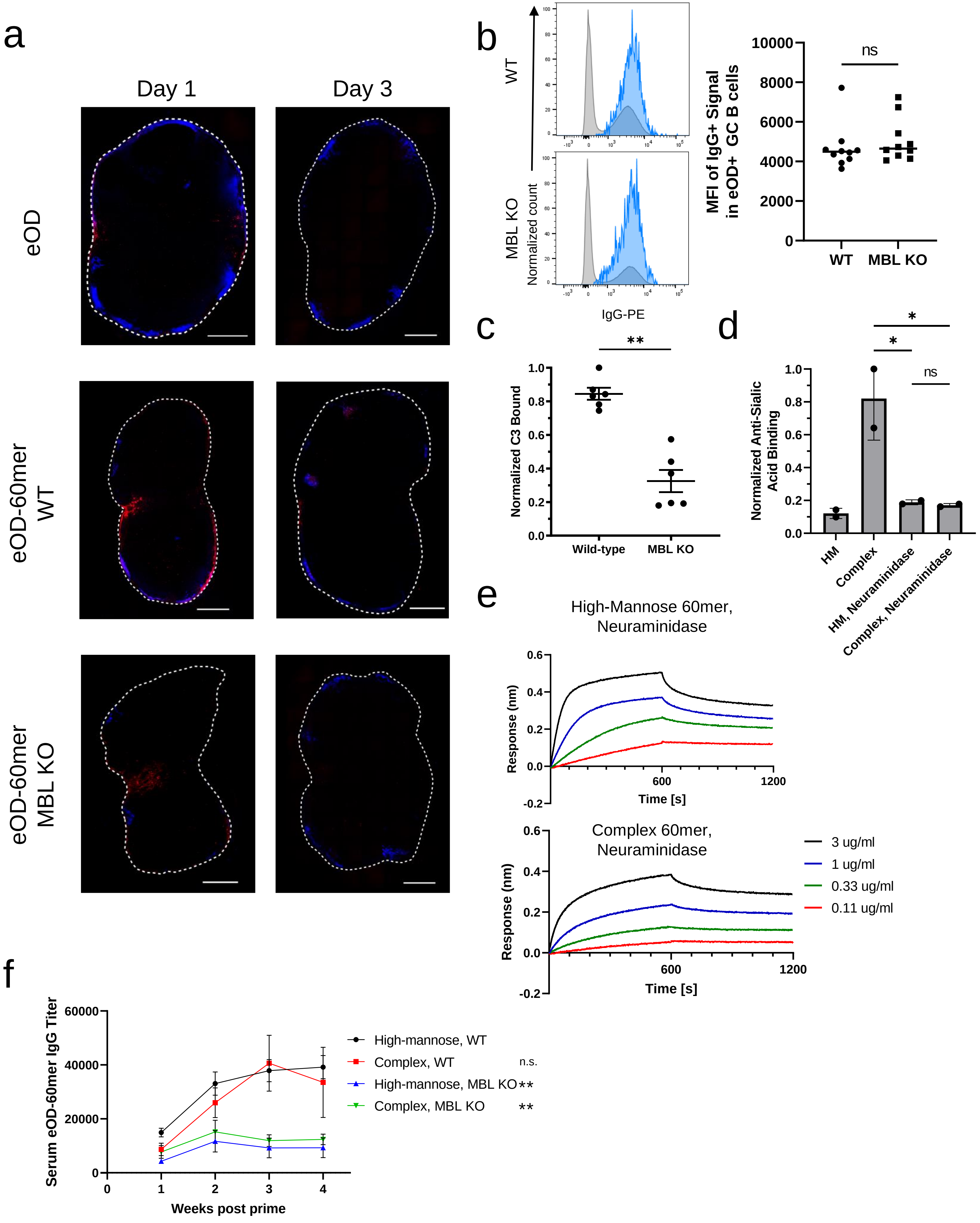

a
Day 1
Day 3
eOD
eOD-60mer WT
eOD-60mer MBL KO
b
WT
MBL KO
Normalized count
IgG-PE
MFI of IgG+ Signal in eOD+ GC B cells
ns
WT
MBL KO
c
Normalized C3 Bound
**
Wild-type
MBL KO
d
Normalized Anti-Sialic Acid Binding
*
*
ns
HM
Complex
HM, Neuraminidase
Complex, Neuraminidase
e
High-Mannose 60mer, Neuraminidase
Complex 60mer, Neuraminidase
Response (nm)
Time [s]
3 ug/ml
1 ug/ml
0.33 ug/ml
0.11 ug/ml
f
Serum eOD-60mer IgG Titer
Weeks post prime
High-mannose, WT
Complex, WT
High-mannose, MBL KO
Complex, MBL KO
n.s.
**
**

Figure S2. Additional lymph node trafficking and characterization of eOD-60mer. Related to Figures 2 and 3. a C57Bl/6 or MBL KO mice (n=5/group) were immunized with 2 µg eOD equivalent AlexaFluor 647-labeled eOD-GT8 60mer or eOD monomer and saponin adjuvant. One or three days post immunization, lymph nodes were snap-frozen and cryosectioned for confocal imaging. Blue, CD35; red, eOD-GT8; scale bars denote 500 µm. **b** C57Bl/6 or MBL KO (n=10/group) were immunized with 2 µg eOD equivalent eOD-GT8 60mer and saponin adjuvant. Draining inguinal lymph nodes were removed 12 days post immunization and analyzed by flow cytometry. Shown are representative histograms of IgG signal MFI among all cells (gray) and antigen-specific GC B cells (blue) and the antigen-specific GC B cell eOD signal MFI from each sample. **c** High-mannose eOD-60mer was incubated for two hours in either 10% fresh wild-type or MBL KO serum (n=6/group), followed by detection with an anti-C3 antibody to determine the degree of C3 deposition. Error bars indicate SEM; **,$p<0.01$ by Mann-Whitney test. **d** High-mannose and complex eOD-60mer were treated with neuraminidase to remove sialic acid residues, which was confirmed via binding of polyclonal anti-sialic acid. n=2/group, *,$p<0.05$ by one-way ANOVA followed by Tukey post hoc test. **e** BLI analysis of high-mannose and complex eOD-60mer treated with neuraminidase binding to murine MBL2 as a function of eOD particle concentration. **f** Serum eOD-60mer specific IgG titers over time in wild-type and MBL KO mice immunized with either high-mannose or complex eOD-GT8 60mer. Error bars indicate SEM; **,$p<0.01$; n.s.=not significant relative to high-mannose WT by one-way ANOVA followed by Tukey post hoc test.

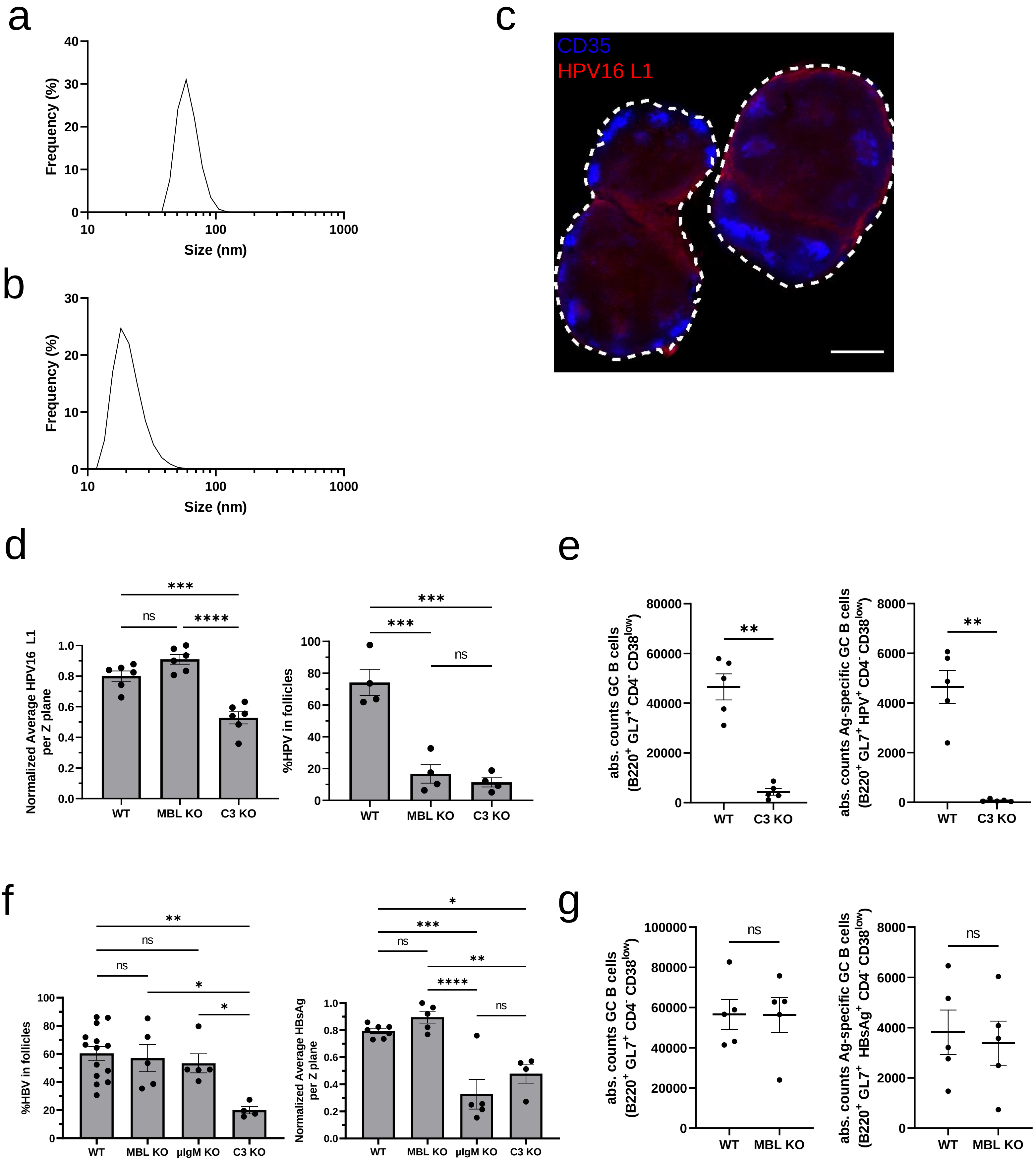
a
Frequency (%)
Size (nm)
b
Frequency (%)
Size (nm)
c
CD35
HPV16 L1
d
Normalized Average HPV16 L1 per Z plane
WT
MBL KO
C3 KO
***
ns
****
%HPV in follicles
WT
MBL KO
C3 KO
***
***
ns
e
abs. counts GC B cells ($B220^+$ $GL7^+$ $CD4^-$ $CD38^{low}$)
WT
C3 KO
**
abs. counts Ag-specific GC B cells ($B220^+$ $GL7^+$ HPV^+ $CD4^-$ $CD38^{low}$)
WT
C3 KO
**
f
%HBV in follicles
WT
MBL KO
μIgM KO
C3 KO
**
ns
ns
*
*
Normalized Average HBsAg per Z plane
WT
MBL KO
μIgM KO
C3 KO
*
***
ns
**
****
ns
g
abs. counts GC B cells ($B220^+$ $GL7^+$ $CD4^-$ $CD38^{low}$)
WT
MBL KO
ns
abs. counts Ag-specific GC B cells ($B220^+$ $GL7^+$ $HBsAg^+$ $CD4^-$ $CD38^{low}$)
WT
MBL KO
ns

Figure S3. Additional HPV16 L1 and HBsAg characterization and *in vivo* response data. Related to Figure 5. a-b Hydrodynamic diameters of HPV16 L1 (**a**) and HBsAg (**b**) as determined by dynamic light scattering. **c** C3 KO mice (n=5/group) were immunized with 0.1 µg AlexaFluor 647-labeled HPV16 L1 and saponin adjuvant. 7 days later, lymph nodes were harvested, cleared, and imaged by confocal microscopy. Shown are average intensity Z projections through 360 µm of tissue; shown is staining for CD35 (blue) and antigen (red), scale bars denote 500 µm. **d** Analyses of normalized total HPV16 L1 signal per Z plane of cleared lymph nodes 7 days post immunization with 0.1 µg AlexaFluor 647-labeled HPV16 L1 and saponin adjuvant from C57Bl/6, MBL KO, and C3 KO mice and percent HPV16 L1 signal found within follicles. Error bars indicate SEM; points represent average values between paired draining lymph nodes from one animal; p***<0.001; p****<0.0001, ns=not significant by one-way ANOVA followed by Tukey post hoc test. **e** C57Bl/6 or C3 KO mice (n=5/group) were immunized with 0.1 µg HPV16 L1 and saponin adjuvant. Absolute counts of germinal center B cells ($B220^+GL7^+CD4^-CD38^{low}$) and antigen-specific germinal center B cells ($B220^+GL7^+$HPV16 $L1^+CD4^-CD38^{low}$) were obtained after 12 days. Error bars indicate SEM, p*<0.05 by Mann-Whitney test. **f** Analyses of normalized total HBsAg signal per Z plane of cleared lymph nodes 7 days post immunization with 5 µg AlexaFluor 647-labeled HBsAg and saponin adjuvant from C57Bl/6, MBL KO, µIgM KO, and C3 KO mice and percent HBsAg signal found within follicles. Error bars indicate SEM; points represent average values between paired draining lymph nodes from one animal; p*<0.05; p**<0.01; p***<0.001; p****<0.0001, ns=not significant by one-way ANOVA followed by Tukey post hoc test. **g** C57Bl/6 or MBL KO mice (n=5/group) were immunized with 5 µg HBsAg and saponin adjuvant. Absolute counts of germinal center B cells ($B220^+GL7^+CD4^-CD38^{low}$) and antigen-specific germinal center B cells ($B220^+GL7^+HBsAg^+CD4^-CD38^{low}$) were obtained after 12 days. Error bars indicate SEM, ns=not significant by Mann-Whitney test.

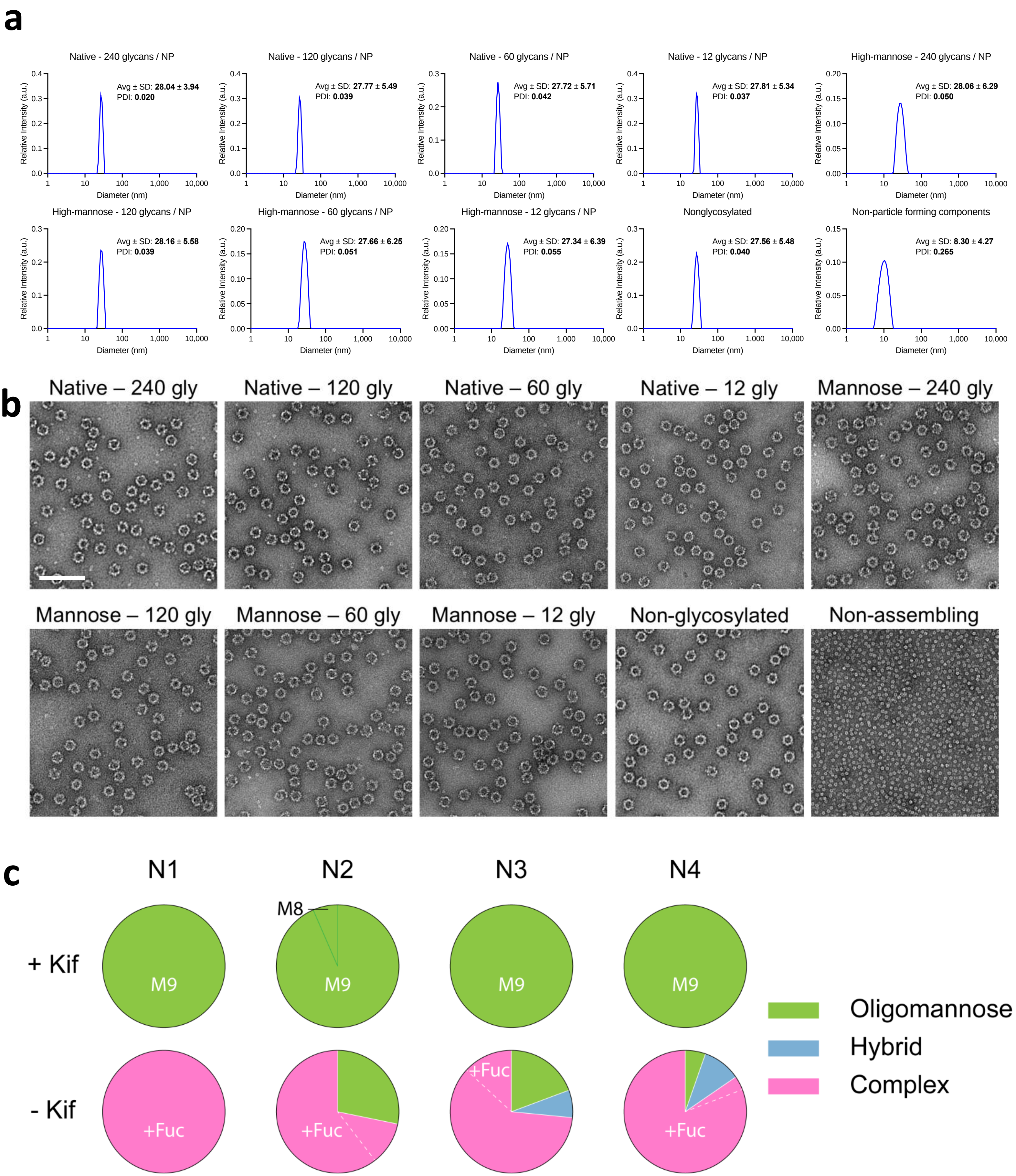
a
Native - 240 glycans / NP
Avg ± SD: 28.04 ± 3.94
PDI: 0.020
Native - 120 glycans / NP
Avg ± SD: 27.77 ± 5.49
PDI: 0.039
Native - 60 glycans / NP
Avg ± SD: 27.72 ± 5.71
PDI: 0.042
Native - 12 glycans / NP
Avg ± SD: 27.81 ± 5.34
PDI: 0.037
High-mannose - 240 glycans / NP
Avg ± SD: 28.06 ± 6.29
PDI: 0.050
High-mannose - 120 glycans / NP
Avg ± SD: 28.16 ± 5.58
PDI: 0.039
High-mannose - 60 glycans / NP
Avg ± SD: 27.66 ± 6.25
PDI: 0.051
High-mannose - 12 glycans / NP
Avg ± SD: 27.34 ± 6.39
PDI: 0.055
Nonglycosylated
Avg ± SD: 27.56 ± 5.48
PDI: 0.040
Non-particle forming components
Avg ± SD: 8.30 ± 4.27
PDI: 0.265
Relative Intensity (a.u.)
Diameter (nm)
1
10
100
1,000
10,000
b
Native – 240 gly
Native – 120 gly
Native – 60 gly
Native – 12 gly
Mannose – 240 gly
Mannose – 120 gly
Mannose – 60 gly
Mannose – 12 gly
Non-glycosylated
Non-assembling
c
N1
N2
N3
N4
+ Kif
- Kif
M8
M9
+Fuc
Oligomannose
Hybrid
Complex

Figure S4. Characterization of glycosylated I53-50 nanoparticles. Related to Figure 6. a Hydrodynamic diameter of glycosylated and non-glycosylated I53-50 nanoparticles and non-assembling control as determined by dynamic light scattering. **b** Negative-stain TEM of glycosylated and non-glycosylated I53-50 nanoparticles and non-assembling control (scale bar, 100 nm). **c** Pie charts of glycan compositions at four N-linked glycosylation sites on I53-50A with and without kifunensine (Kif) treatment (M9: Mannose-9; M8: Mannose-8; +Fuc: Fucosylated glycans).

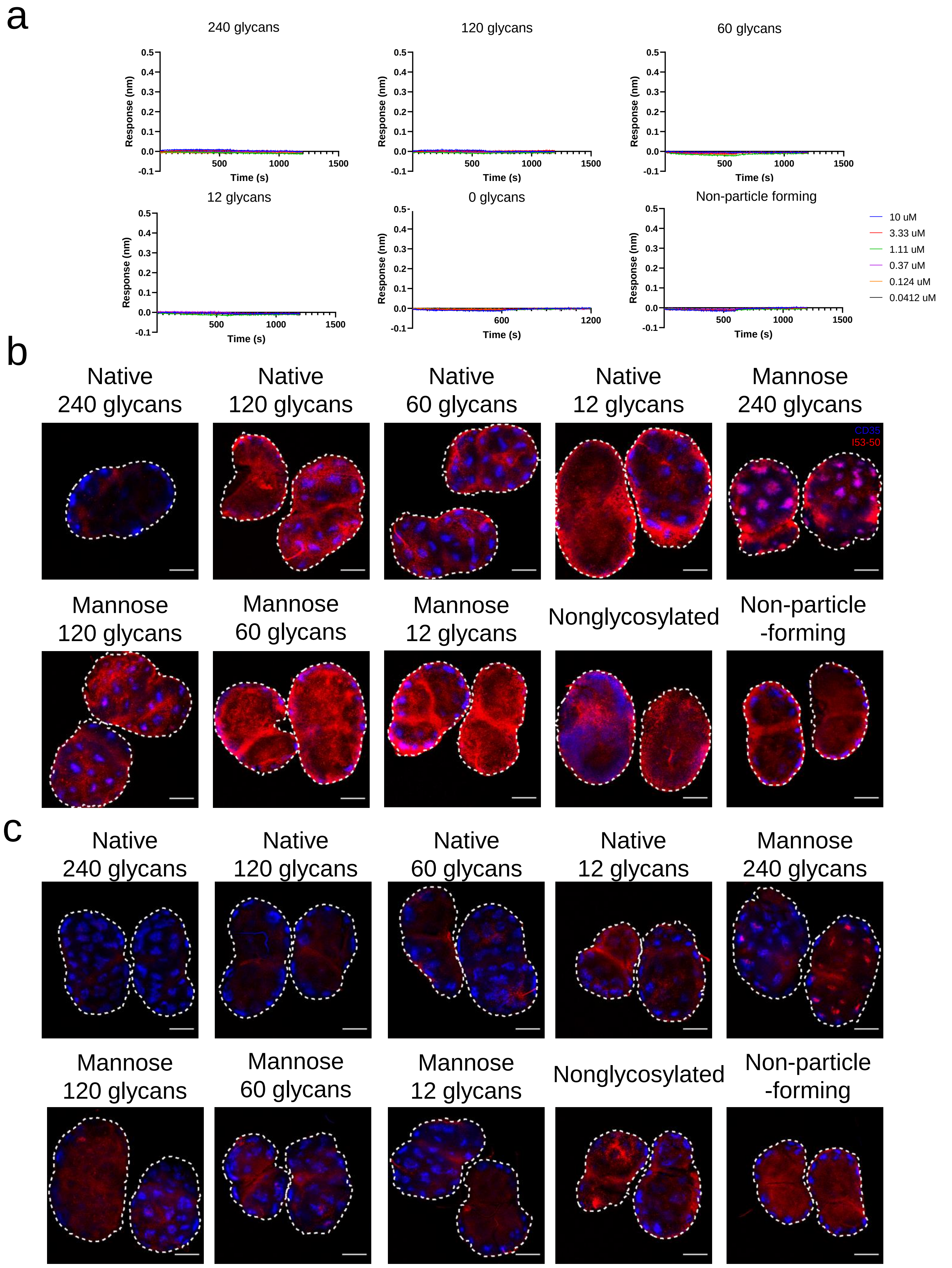

a
240 glycans
120 glycans
60 glycans
12 glycans
0 glycans
Non-particle forming
Response (nm)
Time (s)
10 uM
3.33 uM
1.11 uM
0.37 uM
0.124 uM
0.0412 uM
b
Native 240 glycans
Native 120 glycans
Native 60 glycans
Native 12 glycans
Mannose 240 glycans
CD35
I53-50
Mannose 120 glycans
Mannose 60 glycans
Mannose 12 glycans
Nonglycosylated
Non-particle -forming
c
Native 240 glycans
Native 120 glycans
Native 60 glycans
Native 12 glycans
Mannose 240 glycans
Mannose 120 glycans
Mannose 60 glycans
Mannose 12 glycans
Nonglycosylated
Non-particle -forming

Figure S5. ***In vitro*** **MBL binding and lymph node localization of I53-50 nanoparticles. Related to Figure 6. a** BLI analysis of differentially glycosylated I53-50 nanoparticles binding bearing native, complex glycans to immobilized recombinant murine MBL2 as a function of I53-50 nanoparticle concentration. **b-c** C57Bl/6 mice (n=5/group) were immunized with 5 µg AF647-labeled I53-50 glycan variants and saponin adjuvant. Shown are average intensity Z projections through 360 µm of cleared draining lymph nodes harvested on days 3 (**b**) and 7 (**c**) (blue, CD35; red, I53-50; scale bars denote 500 µm).

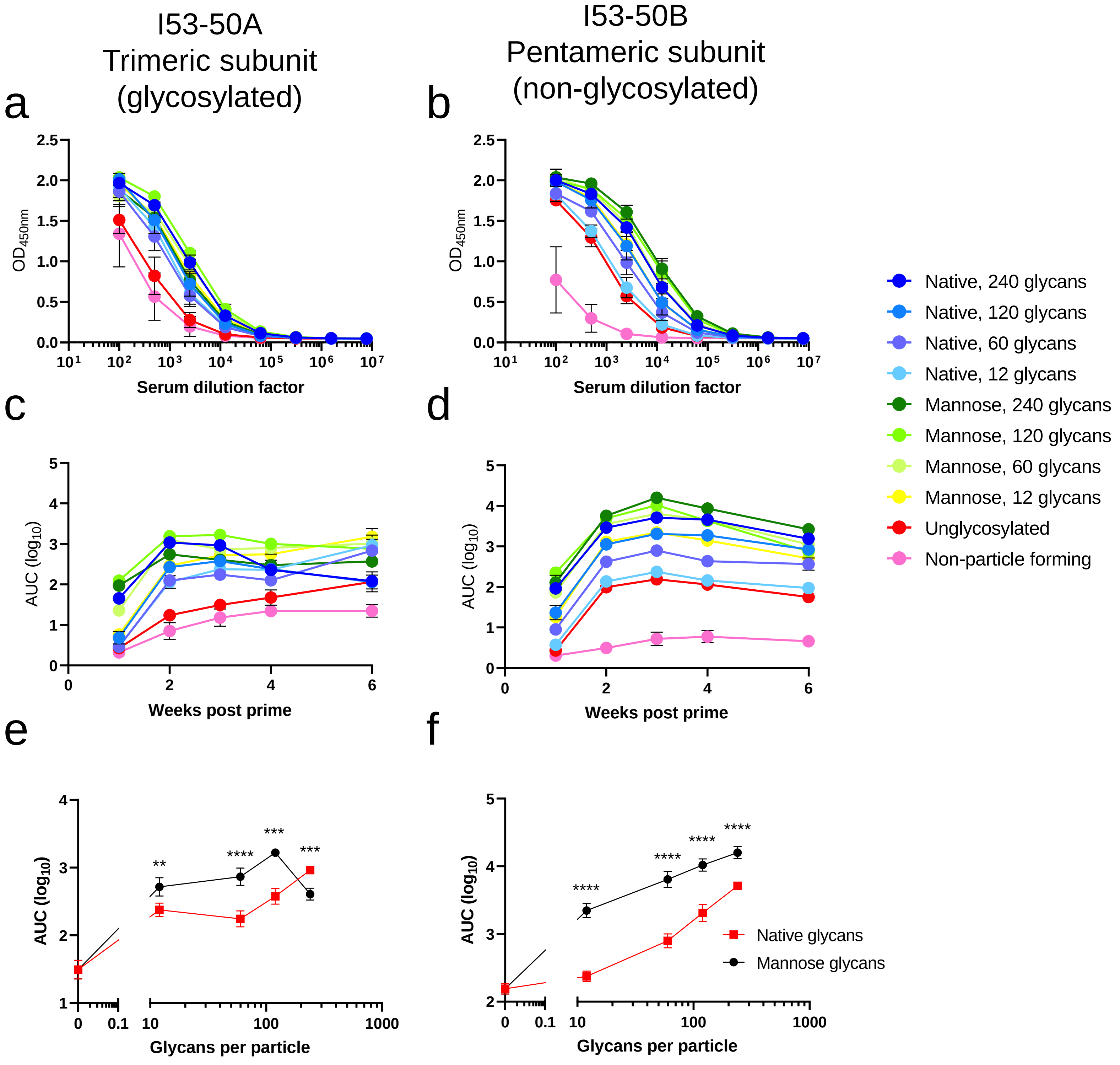

Figure S6. Antibody responses following I53-50 immunization. Related to Figure 6. C57Bl/6 mice (n=5/group) were immunized with 5 µg I53-50 glycan variants and saponin adjuvant. **a-b** Shown are raw ELISA titration curves from three weeks post immunization against I53-50A trimer (**a**) and I53-50B pentamer (**b**) particle subunits. **c-d** AUC measurements of raw ELISA titration curves through six weeks post immunization against I53-50A trimer (**c**) and I53-50B pentamer (**d**) particle subunits. **e-f** Relationship between glycans per particle and AUC of raw ELISA titration curves at three weeks post immunization against I53-50A trimer (**e**) and I53-50B pentamer (**f**) particle subunits. Error bars indicate SEM; p**<0.01; p***<0.001; p****<0.0001 by Mann-Whitney test.
